# Protectin DX reduces inflammatory pain initiated by superoxide anion in mice: targeting leukocyte recruitment, oxidative stress, cytokine production and TRPV1^+^ nociceptive sensory neuron activation

**DOI:** 10.1007/s00011-026-02234-5

**Published:** 2026-04-02

**Authors:** Jessica A. Carneiro, Beatriz H. S. Bianchini, Geovana Martelossi-Cebinelli, Nayara A. Artero, Thaila K. E. Maximiano, Anelise Franciosi, Amanda M. Dionisio, Kelly M. Yaekashi, Thacyana T. Carvalho, Matheus D. V. da Silva, Fernanda S. Rasquel-Oliveira, Janaína Menezes Zanoveli, Victor Fattori, Rubia Casagrande, Waldiceu A. Verri

**Affiliations:** 1 Department of Immunology, Parasitology and General Pathology, Londrina State University, Rodovia Celso Garcia Cid, Km 380, PR 445, Caixa Postal 10.011, Londrina, Paraná 86057-970 Brazil; 2 Department of Biomedical Sciences, Infectious and Immunologic Diseases Research Center, Cedars-Sinai Medical Center, Los Angeles, CA USA; 3 Department of Pharmacology, Biological Science Sector, Federal University of Paraná, Curitiba, Paraná Brazil; 4 Department of Vascular Biology Program, Department of Surgery, Boston Children’s Hospital, Harvard Medical School, Karp Research Building, Boston, MA USA; 5 Department of Pharmaceutical Sciences, Londrina State University, Avenida Robert Koch, 60, Caixa Postal 10.011, Londrina, Paraná 86039-440 Brazil

**Keywords:** Protectin DX, Superoxide anion, Oxidative stress, Pain, Inflammation, TRPV1

## Abstract

**Objective and design:**

This study investigated the antinociceptive potential and mechanisms of Protectin DX (PDX) in a KO_2_-induced inflammatory pain.

**Treatment:**

Male mice received PDX (1, 3, or 10 ng) or vehicle (0.7% ethanol in sterile saline) intraperitoneally (i.p.), 1 h before KO_2_ (30 µg intraplantar [i.pl.] or 1 mg [i.p.]).

**Methods:**

Upon KO_2_ injection, evoked (mechanical and thermal hyperalgesia, and mechanical allodynia) and non-evoked pain behaviors (weight distribution and overt pain-like behaviors), immune cell recruitment (histopathology and immunofluorescence), cytokine production (ELISA), ROS production (NBT assay), antioxidant capacity (ABTS, FRAP, GSH and catalase assays), TRPV1, and neuronal activation (immunofluorescence and calcium imaging) and toxicity parameters (ALT, AST, urea, creatinine and myeloperoxidase assays) were investigated. Motor performance, mechanical and thermal hyperalgesia were also evaluated without the KO_2_ injection.

**Results:**

PDX reduced evoked and non-evoked pain, leukocyte recruitment, production of pro-inflammatory cytokines (TNF-α and IL-1β), and oxidative stress. PDX inhibited TRPV1 activity, resulting in inhibition of nociceptive neuron activation. PDX did not alter the plasma levels of ALT, AST, urea, and creatinine, or stomach myeloperoxidase activity. Also, PDX did not affect the basal mechanical and thermal sensitivity and motor activity.

**Conclusion:**

PDX inhibits superoxide anion-triggered pain and inflammation, through anti-inflammatory, antioxidant, and neuronal component modulation mechanisms.

**Supplementary Information:**

The online version contains supplementary material available at 10.1007/s00011-026-02234-5.

## Introduction

Reactive oxygen species (ROS) are generated mainly through the mitochondrial electron transport chain (ETC), the enzymatic activities of xanthine oxidase (XO), and NADPH oxidase (NOX) [1]. These ROS-generating systems promote oxygen electron reduction, resulting in successful production of superoxide anion, which is an initiating ROS that serves as substrate for the production of hydrogen peroxide (H_2_O_2_), and hydroxyl radical (OH^·^) [2–4]. In addition, the superoxide anion can react with nitric oxide (NO) to form peroxynitrite (ONOO^−^) [4, 5]. In physiological conditions, the actions of ROS are modulated by endogenous enzymatic antioxidant systems, which include, for instance, superoxide dismutase (SOD), catalase, and glutathione peroxidase (GPx) [6, 7]. These enzymes promote the rapid dismutation of highly reactive components into more stable species that are less damaging to cellular components [1].

Inflammatory pain results from the heightened sensitivity and reduced threshold of nociceptive sensory neurons due to exposure to inflammatory mediators produced by immune and tissue cells in inflammatory focus [8]. Superoxide anion contributes to the development of inflammatory pain, as demonstrated by studies using SOD mimetics such as tempol and M40403. The administration of these molecules results in the superoxide anion dismutation to form H_2_O_2_. Consequently, by modulating the redox balance, these pharmacological agents attenuate inflammatory pain [9].

ROS and their oxidation products activate intracellular pathways, such as the nuclear factor-kappa B (NF-κB), mitogen-activated protein kinase (MAPK), and cyclic GMP-AMP synthase—stimulator of interferon genes (cGAS-STING), which results in the production of pro-inflammatory mediators (e.g., IL-1β, TNF-α, IL-6, IL-18, IFN-γ, and PGE_2_) [3, 10–12]. These mediators can sensitize nociceptive neurons resulting in hyperalgesia (an increased response to a stimulus that is normally painful) and/or allodynia (pain due to a stimulus that does not normally provoke pain) [8]. Therefore, recruited leukocytes, mainly neutrophils and macrophages, contribute to ROS production, culminating in nociceptive sensitization [3, 13]. Moreover, the intracellular ROS also activate protein kinases such as protein kinase A (PKA), protein kinase C (PKC), PI3 kinase (PI3K) and MAPK, which phosphorylate ion channels such as transient receptor potential channel ankyrin 1 (TRPA1) and transient receptor potential channel vanilloid 1 (TRPV1) in nociceptive neurons. Kinases such as PI3K and PKA can increase TRPV1 and TRPA1 traffic to the plasma membrane, functional availability, and activity, which contribute to the neuronal activation and sensitization process [14–17]. For example, activation of PI3K signaling leads to the recruitment of Src, which phosphorylates TRPV1 and increases its transport from intracellular compartments, such as the endoplasmic reticulum and Golgi complex, to the plasma membrane, increasing the functional density of TRPV1 channels and contributing to neuronal sensitization [18]. Additionally, the activation of PKA and phospholipase C (PLC) signaling promotes the trafficking of TRPA1 from intracellular vesicular pools to the plasma membrane, thereby increasing the number of neuronal functional channels [17]. These mechanisms increase the functional availability and activity of TRP channels in nociceptive neurons, thereby contributing to neuronal activation and sensitization.

ROS can directly interact with nociceptive neurons, promoting mechanical hyperalgesia and overt pain-like behaviors, for example [19–21]. ROS generate redox alterations on cysteine residues present in the intra- and extracellular portions of ion channels [22–24], such as TRPV1, a channel that is capsaicin-responsive, thermosensitive and widely expressed by nociceptive peripheral C-fibers [25]. This interaction results in channel activation, subsequent influx of Ca^2+^ through the TRPV1 pore, and neuronal activation. Potassium superoxide (KO_2_) is a superoxide anion donor that is used to trigger inflammation, oxidative stress, hyperalgesia, and overt pain-like behaviors dependent on TRPV1, TRPA1, cytokines, cylooxygenase 2 (COX-2), and endothelin-1 (ET-1) [1, 9, 19, 26–28].

Non-steroidal anti-inflammatory drugs (NSAIDs) are widely applied in inflammatory disorders control and pain management in clinical practice. Although effective, these drugs present side effects associated with prolonged use, such as kidney and gastrointestinal damage [29]. In this sense, the research on novel anti-inflammatory drugs persists. Specialized pro-resolving mediators (SPMs) are a class of molecules that actively promote the resolution of inflammation in a time-dependent manner, but they are also anti-inflammatory and non-immunosuppressive [30]. Protectin DX (PDX) (10S,17S-dihydroxy-4Z,7Z,11E,13Z,15E,19Z-docosahexaenoic acid) is a member of the protectin family of SPMs and is biosynthesized from ω-3 fatty acids. This SPM acts on the GPR37 receptor [31] and exerts potent effects in limiting the inflammation response. In lipopolysaccharide (LPS)-induced lung injury models, PDX reduced pulmonary edema, leukocyte recruitment, and the expression of pro-inflammatory mediators (MCP-1, MIP-2, TNF-α, IL-1β, IL-6, and MIP-1α), as well increased IL-10 levels [32, 33]. In a sepsis model, PDX favored bacterial clearance, reduced the levels of pro-inflammatory cytokines, and induced switching towards the pro-resolving macrophage phenotype, conferring improved survival and attenuation of multiple organ damage [34]. Recently, a study showed that PDX down-modulates NLRP3 inflammasome activation in a rheumatoid arthritis model, inhibiting the production of pro-inflammatory cytokines (IL-1β, IL-18, IL-6, TNF-α and IL-17A), increasing the production of anti-inflammatory cytokines (IL-10 and TGF-β), and expanding the population of regulatory T cells, attenuating the inflammatory response in this condition [35]. Different models demonstrated the ability of PDX to trigger antioxidant responses. PDX can control the gene expression of oxidizing and antioxidant factors. PDX administration promotes an increase in the expression of the antioxidant enzymes SOD2 and catalase [36], and reduces inducible nitric oxide synthase (iNOS) expression [37]. In addition, PDX inhibits the phosphorylation of the p47^phox^ subunit of the NOX2 multiprotein complex, thereby attenuating ROS production, and decreasing the nitric oxide (NO) levels [37, 38]. Consequently, PDX administration results in reduced lipid peroxidation (LPO), as demonstrated by reduced malondialdehyde (MDA) levels [39].

Evidence demonstrates that PDX has anti-inflammatory effects and the ability to induce antioxidant responses both in vitro and in vivo models. However, its antinociceptive effect and mechanisms on acute inflammatory pain has not been consistently explored. Therefore, we aimed to investigate the PDX effects and mechanisms on pain and inflammation superoxide anion-induced in mice.

## Materials and methods

### Animals

Male Swiss mice from the State University of Londrina, Paraná, Brazil, or male LysM-eGFP C57BL/6 background mice from the Ribeirão Preto Medical School, São Paulo, Brazil, weighing between 20 and 25 g, were used in this study. Mice were housed in standard clear plastic cages with water and food provided ad libitum, in a light/dark cycle of 12/12 h, controlled temperature (22 ± 1 °C) and air exhaust. Mice were maintained in the vivarium of the Department of Immunology, Parasitology, and General Pathology of State University of Londrina for at least two days before experiments. Mice were used only once and were acclimatized to the testing room, in a variable time depending on the method, before the experiments, which were conducted during the light cycle. Animals that were within the weight range and not previously injured were used. In addition, the mice were randomly assigned to the groups. The randomization method employed involved the distribution of mice randomly assigned to groups by manual lottery. Randomization was performed by a laboratory technician not involved in data collection. The sample size was determined using G*Power software. Euthanasia was performed by three sequential procedures to minimize animal suffering. First, mice were anesthetized with a lethal dose (5%) of isoflurane, followed by cervical dislocation, and subsequently decapitation. Animal care and handling procedures were in accordance with the ARRIVE 2.0, the International Association for Study of Pain (IASP) guidelines and approved by the State University of Londrina Ethics Committee Animal Research and Welfare (process number 003.2021). All efforts were made to minimize the number of mice used and their suffering. A total of 513 mice were used for all the experiments.

### Experimental procedures

**Fig. 1:**
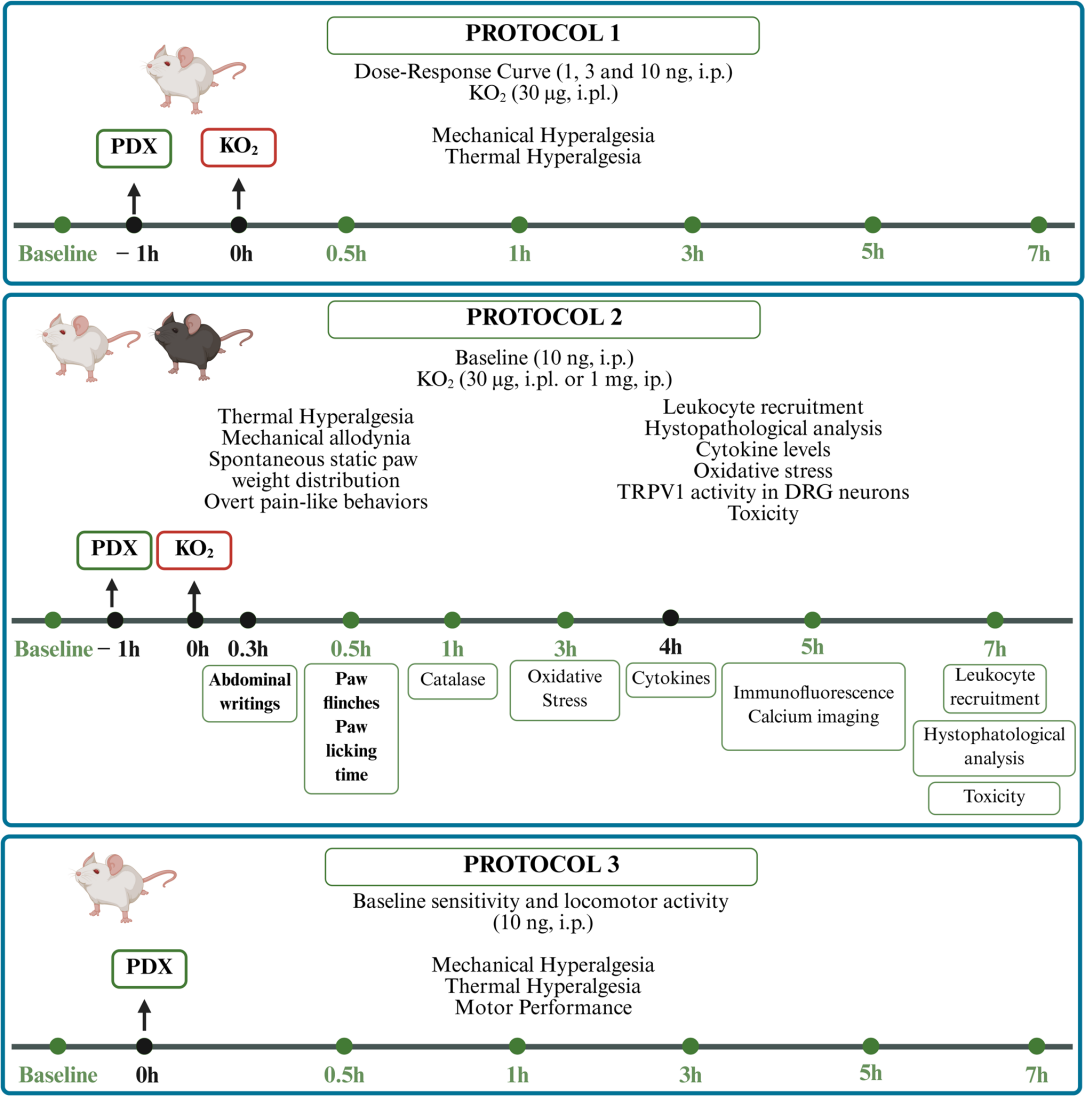
Experimental design. Protocol 1, mice were pretreated with PDX (1, 3, or 10 ng, i.p.) or vehicle (0.7% ethanol in sterile saline, NaCl 0.9%, 100 µL, i.p.) 1 h before KO_2_ (30 µg, 20 µL/paw, i.pl.) or vehicle (sterile saline, NaCl 0.9%, 20 µL/paw, i.pl.) injection. The dose–response curve was determined by analyzing the parameters of mechanical hyperalgesia and thermal hyperalgesia determined by electronic von Frey and hot plate, respectively, were evaluated 0 (baseline), 0.5, 1, 3, 5, and 7 h after the KO_2_ injection. PDX (10 ng) was selected for the subsequent experiments. Protocol 2 includes analysis of additional pain parameters, leukocyte recruitment, cytokine production, oxidative stress, TRPV1 channel activity in DRG neurons, and toxicity. Thermal hyperalgesia (Hargreaves test), mechanical allodynia (von Frey filaments) and weight distribution on the hind paws (static weight bearing) were assessed 0 (baseline), 0.5, 1, 3, 5, and 7 h after the KO_2_ injection. The overt pain-like behaviors evaluated were abdominal writhings, paw flinches, and paw licking time. Exceptionally, for the abdominal writhings, the mice received a KO_2_ i.p. injection (1 mg, 100 µL/peritoneal cavity), and the number of abdominal writhings was counted over 20 min. The paw flinches and licking time were counted over 30 min after the KO_2_ i.pl. injection (30 µg, 20 µL/paw). The hind paw skin was collected at 1, 3, 4, and 7 h after the stimulus to assess the parameters of oxidative stress (catalase, GSH, FRAP, ABTS, and NBT assays), cytokine production, and leukocyte recruitment, respectively. To evaluate the TRPV1 activity, DRG neurons were dissected 5 h after KO_2_ injection for the immunofluorescence and calcium imaging assays. Stomach and plasma samples were collected 7 h after the stimulus to assess toxicity parameters. At last, Protocol 3 described the evaluation of PDX treatment on motor activity and basal mechanical and thermal sensitivities. In the absence of KO_2_ injection, mice were pretreated with PDX (10 ng, 100 µL, i.p.) or vehicle (0.7% ethanol in sterile saline, NaCl 0.9%, 100 µL, i.p.). Mechanical hyperalgesia was evaluated by electronic von Frey, at times 0 (baseline), 0.5, 1, 3, 5, and 7 h after the treatment. Thermal hyperalgesia, determined by hot plate and Hargraves test, at times 0 (baseline), 1, 3, 2 5 and 7 h after the treatment. Motor function was evaluated using the rotarod performance test at times 0 (baseline), 0.5, 1, 3, 5 and 7 h after the treatment. Created in BioRender. Oliveira (2025) (https://BioRender.com/yna52dl, accessed 14th June 2025)

Firstly, male Swiss mice were pretreated intraperitoneally (i.p.) with vehicle (0.7% ethanol in sterile saline, NaCl 0.9%, 100 µL) or PDX (1, 3, or 10 ng, diluted in saline, 100 µL) 1 h before intraplantar (i.pl.) vehicle (sterile saline, NaCl 0.9%, 20 µL) or KO_2_ (30 µg, diluted in saline, 20 µL) injection. All tests were performed by injecting the KO_2_ in the paw except by the abdominal writhings test for which the KO_2_ in the peritoneum (1 mg, 100 µL, i.p.). Mice were acclimated to the measuring equipment for 3 consecutive days before the start of the experiments. On the day of the experiment, the baseline was measured at one time point before stimulation with KO_2_. The dose–response curve was determined using mechanical and thermal hyperalgesia parameters (Fig. 1, Protocol 1). Mechanical hyperalgesia, measured by electronic von Frey, and thermal hyperalgesia, determined by Hot Plate Test, were evaluated at the following times: 0 (baseline before any injection), 0.5, 1, 3, 5, and 7 h after KO_2_ or vehicle injection. The dose of PDX of 10 ng/mouse was selected for the subsequent experiments.

Next, Protocol 2 was developed, which includes analysis of additional pain parameters, leukocyte recruitment, cytokine production, oxidative stress, TRPV1 channel activity in DRG neurons, and toxicity (Fig. 1, Protocol 2). Thermal hyperalgesia (Hargreaves test), mechanical allodynia (von Frey filaments), and unbalanced weight distribution between the hind paws (static weight bearing apparatus) were evaluated at times 0 (baseline), 0.5, 1, 3, 5, and 7 h after KO_2_ or vehicle injection. The overt pain-like behaviors were assessed by the number of abdominal writhings, flinches and licking time after stimulus expressed cumulatively over 20 min and 30 min, respectively. The neutrophil and macrophage recruitment to the paw skin were evaluated 7 h after KO_2_ injection (H&E staining and LysM-eGFP fluorescence measurement). Only for LysM-eGFP fluorescence C57BL/6 background mice were used because of GFP reporter of lysozyme expression characteristic of neutrophils and macrophages, otherwise, Swiss mice were used. Cytokine levels (TNF-α and IL-1β) in hind paw skin were performed 4 h after KO_2_ injection by ELISA assay. Oxidative stress assays were performed 1 (catalase assay) and 3 h (FRAP, ABTS, NBT, and GSH assays) after KO_2_ injection, reflected by the measurements of antioxidant defenses and superoxide anion production. TRPV1 activity in DRG was assessed by immunofluorescence and calcium imaging method 5 h after KO_2_ injection. We evaluated whether acute treatment with PDX (10 ng, i.p.) could cause gastric, hepatic, or renal damage. Stomach and plasma samples were collected 7 h after the KO_2_ injection, to determine stomach myeloperoxidase (MPO) activity and plasma concentrations of aspartate aminotransferase (AST), alanine aminotransferase (ALT), urea, and creatinine. In these toxicity assessment tests, positive control groups were treated with indomethacin (2.5 mg/kg, i.p., sampling within seven days) for gastric injury, acetaminophen (650 mg/kg, orally, sampling within 24 h) for liver injury, or diclofenac (200 mg/kg, orally, sampling within 24 h) for kidney injury [40–45]. Finally, we evaluated the baseline mechanical and thermal sensitivity and locomotor activity, in the absence of KO_2_ injection (Fig. 1, Protocol 3). Mice were pretreated (1 h) with PDX (10 ng, 100 µL) or vehicle (0.7% ethanol in sterile saline, NaCl 0.9%, 100 µL). Then, measurements of mechanical hyperalgesia (electronic von Frey), at times 0 (baseline), 0.5, 1, 3, 5, and 7 h after the treatment, and thermal hyperalgesia (hot plate and Hargreaves test), at times 0 (baseline), 1, 3, 5 and 7 h after the treatment. In addition, the motor function was evaluated using the rotarod performance test, at times 0 (baseline), 0.5, 1, 3, 5, and 7 h after the PDX treatment. The assessment of responses upon PDX in the absence of KO_2_ stimulation allowed concluding whether this SPM has antinociceptive effects (would not alter basal responses) or would impair the behavioral responses in basal conditions.

### Drugs, reagents, and antibodies

Drugs and reagents used in this study were obtained from the following sources:

Protectin DX (10(S), 17(S)-DiHDHA) was from Cayman Chemical (Ann Arbor, Michigan, USA); superoxide anion (KO_2_) 96.5% was from Alfa Aesar (Ward Hill, Massachussetts, USA) and saline solution (NaCl 0.9%) was from Fresenius Kabi Brazil Ltda. (Aquiraz, Ceará, Brazil). ELISA kits for measurement of TNF-α and IL-1β were from eBioscience (Thermo Fisher Scientific, VIE, Austria). Hank's Balanced Salt Solution (HBSS) was from Thermo Fisher Scientific (Waltham, MA, USA). RPMI medium was from (Thermo Fisher Scientific, VIE, Austria). Neurobasal-A medium (NBM) was purchased from Life Technologies (Thermo Fisher Scientific); Collagenase A (RocheApplied Sciences, Indianapolis, Indiana, USA), Dispase II was from Roche Applied Sciences (Indianapolis, IN, USA); 4-(2-hydroxyethyl)-1-piperazine ethane sulfonic acid (HEPES)-buffered saline was from Millipore Sigma (Burlington, MA, USA); and Fluo‐4 a.m. was from Invitrogen (#F14201, Carlsbad, CA, USA). Isoflurane was from Abbott Laboratories (Abbott Park, Illinois, USA); Nitroblue tetrazolium (NBT) was from Amresco (Solon, Ohio, USA); ferric chloride hexahydrate, 2,4,6- tripyridyl-s-triazine (TPTZ) and ABTS (2,20-azino-bis(3-ethylbenzothiazoline-6-sulfonate)) was from Sigma-Aldrich (St. Louis, Missouri, USA). The fluorescent antibodies were: anti-capsaicin receptor antibody (#ab5566; Guinea pig, Merck Millipore, Burlington, Massachusetts, USA), anti-phosphorylated NF-κB p65 (#sc-136548; Mouse, Santa Cruz Biotechnology, Dallas, TX, USA), anti-Guinea pig secondary antibody (Alexa Fluor 488- Goat, #A11073, Thermo Fisher Scientific, Waltham, MA, USA) and anti-mouse secondary antibody (Alexa Fluor 647-Goat, #115-605-003; Jackson ImmunoResearch, West Grove, PA, USA). 4′,6-Diamidine-2′-phenylindole dihydrochloride (DAPI) was from Thermo Fisher Scientific (Waltham, MA, USA).

### Mechanical hyperalgesia

The von Frey filaments electronic version test was used to evaluate plantar cutaneous hyperalgesia. In a quiet, temperature-controlled room, mice were placed in an acrylic compartment (12 × 10 × 17 cm) with a wire grid floor, 1 h before the start of testing. The tests consisted of evoking a hind paw flexion reflex with a handheld force transducer (electronic aesthesiometer, IITC Life Science, Woodland Hills, CA) adapted with a 0.5 mm^2^ polypropylene tip. The investigator was trained to apply the tip perpendicularly to the central area of the plantar hind paw with a gradual increase in pressure. The endpoint was characterized by the removal of the paw by clear flinching or licking movements. After paw withdrawal, the intensity of the pressure was automatically recorded, with the final response value obtained by averaging the results of two measurements. Mice were acclimated for 3 consecutive days prior to the experiment, remaining in the equipment for 1 h in each day. Baseline values were obtained prior to KO_2_ injection in the same day in triplicate. The results are expressed by the delta (∆) withdrawal threshold (in grams), which was calculated by subtracting the mean measurements at 0.5–7 h after KO_2_ injection or PDX administration from the zero-time (baseline values) mean measurements. PDX dose–response was measured using 6 mice/group (*N* = 30). To assess mechanical sensitivity, 6 mice/group (*N* = 12) were used. The investigators were blinded to the treatment.

### Thermal hyperalgesia

Plantar cutaneous thermal hyperalgesia was assessed using the Hargreaves Test and Hot Plate Test as previously described [19, 46]. In the hot plate test, the mice were placed on a metal plate with a constant temperature (52 °C ± 1 °C) (Insight, Ribeirão Preto, São Paulo, Brazil), and the endpoint was characterized by the removal of the paw followed by clear hind paw flinching or licking movements. The results are expressed by withdrawal latency (in seconds). In the Hargreaves test, mice were individually confined to plexiglass chambers, and a high-intensity projector bulb was positioned beneath the right hind paw to a direct thermal stimulus. Mice were acclimated for 3 consecutive days prior to the experiment, remaining in the equipment for 1 h in each day. Baseline values were obtained prior to KO_2_ injection in the same day in triplicate. The withdrawal latency period was recorded using an electronic timer circuit. For both tests, the results were expressed as the average of two measurements and are expressed by withdrawal latency (in seconds). In both tests, a cut-off of 20 s was set to avoid tissue damage. PDX dose–response was measured using 6 mice/group (*N* = 30). Thermal hyperalgesia measured by Hargreaves test, was applied 6 mice/group (*N* = 18). To assess thermal sensitivity by hot plate, 6 mice/group (*N* = 12) were used. To assess thermal sensitivity by Hargreaves test, 6 mice/group ( *N =* 12) The investigators were blinded to the treatment.

### Mechanical allodynia

Mechanical allodynia was evaluated by a manual von Frey filaments test. In a quiet, temperature-controlled room, mice were placed in an acrylic compartment (12 × 10 × 17 cm) with a wire grid floor, 1 h before the start of testing. The tests consisted of evoking a hind paw flexion reflex with a series of filaments (0.04, 0.07, 0.016, 0.4, 0.6, 1, 1.4, 2, and 4 g) applied in sequence to start with one that possessed a bucking weight of 0.6 g, was applied in sequence to the plantar surface of the ipsilateral paw with a pressure that caused the filament to buckle. The hind paw flexion reflex was recorded as a positive response and the next lightest filament was chosen for the subsequent measurement. The absence of a response prompted the use of the next filament of increasing weight. The 50% withdrawal threshold was determined using the “up-down” method, which requires four stimuli straddling the threshold. Mice were acclimated for 3 consecutive days prior to the experiment, remaining in the equipment for 1 h in each day. Baseline values were obtained prior to KO_2_ injection in the same day in triplicate. The results were expressed as a single measurement per time point (baseline, 1, 3, 5, and 7 h after KO_2_ injection). Baseline values were obtained prior to KO_2_ injection on the same day as the other measurements. To assess mechanical allodynia, 6 mice/group (*N* = 18) were used. The investigators were blinded to the treatment.

### Overt pain-like behavior

The overt pain-like behaviors evaluated were abdominal writhing, flinches, and paw licking time. To assess the abdominal writhing, mice were gently and individually placed in a glass cylinder with enough space for free movement. In this test, a positive response was considered when the mice performed a stretching of the hind limbs associated with a slow abdominal wall contraction. The number of abdominal writhings was assessed cumulatively over 20 min. The total number of paw flinches and time spent licking the paw were counted 30 min after the KO_2_ injection. Results were expressed as the total number of abdominal writhings, flinches, or time spent licking the paw (in seconds) performed in 20 or 30 min. The investigators were blinded to the treatment. For flinch and licking measurements, 10 mice/group were used (*N* = 30). For abdominal writhing measurement, 6 mice/group were used (*N* = 18). The investigators were blinded to the treatment.

### Static weight bearing

Unilateral peripheral inflammation produces changes in paw weight distribution toward the non-injured paw [47]. Alterations in paw weight distribution were evaluated using the SWB apparatus (BIO-SWB-TOUCH-M, Bioseb, France). In a quiet, temperature-controlled room, mice were placed into an acrylic chamber, where the mice were comfortably maintained while their hind paws rested on two separate sensor plates. The mice stand and make a natural adjustment to the degree of pain by adapting weight distribution on the non-injured hind paw, and the weight value applied on each sensor is displayed on the LCD screen of the control unit. Mice were acclimated, individually, for 3 consecutive days prior to the experiment, remaining in the equipment for 5 min in each day. Baseline values were obtained prior to KO_2_ injection in the same day in triplicate. Results were expressed by the right/left paw ratio, which was calculated by using the mean of two measurements. The results were expressed as the average of two measurements. To assess weight distribution, 6 mice/group (*N* = 18) were used. The investigators were blinded to the treatment.

### Rotarod test

The rotarod test was used to evaluate whether treatment with PDX affects the motor function of mice. The apparatus consists of a bar 2.5 cm in diameter, subdivided into four compartments by disks of 25 cm in diameter (Ugo Basile, model 7600). During the measurements, the bar rotates at a constant speed of 22 rotations per minute. Mice were acclimated for 3 consecutive days prior to the experiment, remaining in the equipment for 3 min in each day. Baseline values were obtained prior to KO_2_ injection in the same day in triplicate. The results were expressed as the average of two measurements. Results were expressed in seconds. The cut-off time used was 180 s. To assess motor function, 6 mice/group (*N* = 12) were used. The investigators were blinded to the treatment.

### Paw tissue histology

Mice plantar tissue was collected 7 h after KO_2_ or vehicle injection. Paraffin-embedded hind paw tissue was processed for hematoxylin and eosin (H&E) staining. Paw samples were fixed with 10% paraformaldehyde before embedding. The sectioned tissues (7 µm) were stained with HE for later examination under an optical microscope (Olympus OX31, Olympus, Japan; original magnification, 40×). The clinical score evaluated the parameters of neovascularization, inflammatory infiltration, and tissue degradation. The scores ranged from 0 to 3, with 0 for no change and 3 for the most serious change, in which case it could be a significant increase in new vessels and inflammatory infiltrate that damage the tissue. Analyzes were performed on ImageJ 1.44 software for Windows (Java image software in the public domain: 14 http://rsb.info.nih.gov/ij/) using the threshold tool and performed on RGB images without further treatment. Three non-consecutive cuts per animal were obtained. Six non-overlapping fields per animal were evaluated, using fields from the central portion of the plantar tissue. The individual result for each animal was obtained by averaging the six fields of each one of the three analyzed slices. Leukocyte recruitment was determined using 86,144 µm^2^ as the dimension area. Each group consisted of 4 animals (*N* = 12).

### Paw tissue fluorescence

LysM-eGFP C57BL/6 mice plantar tissue was collected 7 h after KO_2_ or vehicle injection. LysM-eGFP C57BL/6 mice express enhanced green fluorescent protein (eGFP) expression controlled by the lysozyme M promoter (LysM) present in neutrophils and macrophage granules. Plantar samples were maintained in 4% 26 paraformaldehyde (PFA, 24 h) and then in 30% sucrose (72 h). Then hind paws were embedded in optimum cutting temperature (Tissue-Tek 1, O.C.T. Compound, IA018, ProSciTech, Australia), and 30 µm sections that were cut in a cryostat and processed for immunofluorescence. The nuclei were counterstained with DAPI (4’,6-diamidino2-phenylindol (1:500, cat #14,285, Cayman Chemicals, Ann Arbor, MI, USA). Imaging was performed using a confocal microscope (Leica TCS SP8, Leica, Wetzlar, Germany) with a 40 × objective. Images were processed using Leica EL6000 software (Leica, Wetzlar, Germany). Three step sections per animal were obtained. Six non-overlapping random fields per animal were evaluated, using fields from the central portion of the plantar tissue. The fluorescence intensity was quantified by an investigator blinded to the treatment in randomly selected fields (six fields per mice, n = 10) of different groups as an indication of neutrophil/macrophage recruitment to the paw tissue. The results were expressed as eGFP fluorescent intensity. Each group consisted of 10 animals (*N* = 30). The fluorescence intensity was quantified by an investigator blinded to the treatment.

### Cytokine measurement

Mice paw skin tissue was collected 2 h after KO_2_ or vehicle injection. Each paw was processed individually. The plantar tissue was fragmented into four small pieces using a surgical scalpel blade by performing one vertical and one horizontal cut across the center of the specimen. The fragments of one animal then placed into a single well of a 24-well plate containing 300 µL of DMEM (Dulbecco’s Modified Eagle’s Medium, Sigma-Aldrich, St. Louis, Missouri, USA), with one paw, divided into four pieces incubated per well. Samples were incubated in a humidified incubator (37 °C, 5% CO_2_) for 2 h. After the incubation period, the culture supernatant was collected for cytokine quantification. Thus, cytokine levels were determined 2 h after KO_2_ or vehicle injection plus 2 h of incubation (totaling 4 hours). IL-1β and TNF-α concentrations were measured by enzyme-linked immunosorbent assay (ELISA) using commercially available kits (eBioscience, San Diego, CA, USA), according to the manufacturer’s instructions. Absorbance was read at 450 nm. Results were expressed as picograms (pg) per mL of culture medium for each cytokine. Each group consisted of 10 animals (*N* = 30).

### Reduced glutathione (GSH) levels

Mice paw skin tissue was dissected 3 h after i.pl. KO_2_ or vehicle injection. Homogenates were mixed with 25 µL of 50% trichloroacetic acid, vortexed three times for 15 min, and centrifuged (15 min, 1500 g, 4 °C). In the supernatant was added to 200 µL Tris buffer 0.2 M, pH 8.2, and 10 µL 5,5’-dithiobis(2-nitrobenzoic acid) (DNTB) 0.01 M. After 5 min of incubation at room temperature, the absorbance was measured at 412 nm. A standard curve of GSH was used, and the results are expressed as GSH (nmol per mg of tissue). Each group consisted of 10 animals (*N* = 30).

### Catalase levels

Catalase activity was measured by the decay in the H_2_O_2_ concentration. Mice paw skin tissue was dissected 1 h after i.pl. KO_2_ or vehicle injection. Samples were homogenized in 500 µL of 0.02 M EDTA using a Tissue-Tearor (Biospec), and centrifuged twice (200 g, 10 min, 4 °C). The reaction mixture contained 10 µL of the sample, 160 µL of buffer Tris–HCl 1 M with EDTA 5 mM (pH 8.0), 20 µL of deionized water, and 20 µL of H_2_O_2_ 200 mM. Catalase activity was determined through the difference between the initial reading and the reading conducted 30 s after the addition of H_2_O_2_ at 240 nm in a microplate reader (EnSpire, Perkin Elmer) at 25 °C. The catalase values were expressed as units of catalase/mg of tissue/minute. Each group consisted of 10 animals (*N* = 30).

### Superoxide anion production

Mice paw skin tissue was dissected 3 h after i.pl. KO_2_ or vehicle injection. Nitroblue tetrazolium assay was used to determine superoxide anion production. Samples were incubated (25 °C, 1 h) with an NBT reagent (100 µL, 1 mg/mL). The supernatant was removed by pipetting, and the reduced formazan was solubilized with 120 µL of potassium hydroxide (KOH) and 140 µL Dimethyl Sulfoxide (DMSO). The measurement was performed at 600 nm using a microplate spectrophotometer reader (Multiskan GO, Thermo Fisher Scientific). The results are expressed as NBT reduction (OD/mg of tissue). Each group consisted of 10 animals (*N* = 30).

### Total antioxidant capacity

Mice paw skin tissue was dissected 3 h after i.pl. KO_2_ or vehicle injection. The capacity to counteract oxidative deleterious effects was evaluated for Ferric Reducing Ability Potential (FRAP) and Ability to Scavenge the 2′2’-Azino-bis(3-ethylbenzothiazoline-6-sulphonic Acid) (ABTS) Radical assays. Samples were homogenized in KCl buffer, for subsequent centrifugation (200 g × 10 min × 4 °C). The supernatant was used for both FRAP and ABTS tests. In the FRAP assay, samples were incubated (25 °C, 30 min) with 150 µL FRAP reagent and read at 595 nm (Multiskan GO, Thermo Fisher Scientific, Vantaa, Finland). In the ABTS scavenging, samples were incubated (25 °C, 6 min) with 200 µL ABTS and read at 730 nm. A standard Trolox curve (0.02–20 nmol) was used to equalize FRAP and ABTS tests. Results were presented as nanomoles of Trolox equivalent/milligram of tissue. Each group consisted of 10 animals (*N* = 30).

### Immunofluorescence staining

DRG segments from region L4-L6 were dissected 5 h after KO_2_ or vehicle injection. Mice were first intracardially perfused with PBS followed by 4% PFA. DRG samples were then post-fixed with 4% PFA overnight at 4 °C and then replaced with 30% sucrose. DRG was embedded in an OCT compound, and 10 µm sections were cut in a cryostat and processed for immunofluorescence. Before incubation with antibodies, slides were blocked with 5% goat serum in BS, 0.5% Triton X-100 (0.5% PBST). Then, sections were incubated with primary antibodies anti-TRPV1 (1:500, #ab5566, Guinea pig, Merck Millipore, Burlington, Massachusetts, USA) and anti-phosphorylated NF-κB p65 (#sc-136548; Mouse, Santa Cruz Biotechnology, Dallas, TX, USA). The nuclei were marked with DAPI (4’,6-diamidino2-phenylindol (1:500, cat #14,285, Cayman Chemicals, Ann Arbor, MI, USA). All primary antibodies and DAPI were incubated overnight at 4 °C (2% goat serum in 0.5% PBST) and then washed four times with 0.5 PBST before proceeding with secondary antibody incubations. After that, sections were stained with secondary antibody Alexa Fluor 488 goat anti-Guinea pig (1:500, Guinea-pig, #A11073, Thermo Fisher Scientific, Waltham, MA, USA) and Alexa Fluor 647 goat anti-mouse (1:500, Mouse, #115-605-003; Jackson ImmunoResearch, West Grove, PA, USA). All secondary antibodies were incubated for 60 min at room temperature (2% goat serum in 0.5% PBST). Stained slides were then washed five times with 0.3% PBST and mounted in Fluoromount-G compound (cat #0100-01, SouthernBiotech, Birmingham, AL, USA). The images were taken in a Confocal microscope (TCS SP8, Leica Microsystems, Mannheim, Germany) and fluorescence intensity was measured using Leica EL6000 software (Leica, Wetzlar, Germany). One field per animal was evaluated. Of note, an entire DRG can be analyzed in one field. Results are expressed in the percentage of TRPV1^+^ neurons, pNF-κB^+^ neurons, and TRPV1^+^/pNF-κB^+^ neurons. Each group consisted of 10 animals (*N* = 30).

### Calcium imaging

DRGs were dissected into Neurobasal-A medium (Life Technologies, Thermo Fisher Scientific, Waltham, Massachusetts, USA), dissociated in collagenase A (1 mg/mL)/dispase II (2.4 U/mL) (RocheApplied Sciences, Indianapolis, Indiana, USA) in HEPES-buffered saline (MilliporeSigma, Burlington, Massachusetts, USA) for 70 min at 37 °C. After trituration with glass Pasteur pipettes of decreasing size, DRG cells were centrifuged over a 10% BSA gradient, plated on laminin-coated cell culture dishes. DRGs were loaded with 1.2 µM of Fluo-4AM (Invitrogen, Carlsbad, California, USA) in Neurobasal-A medium, incubated for 30 min 37 °C, washout with HBSS and imaged in Confocal Microscope (TCS SP8, Leica Microsystems, Mannheim, Germany). To evaluate activation, DRG plates were recorded for 6 min, which was divided in: 2 min of initial reading (0 s mark, baseline values), following by stimulation with capsaicin for 2 min at 120 s mark (1 µM, TRPV1 agonist) and KCl for 2 min at 240 s mark (40 mM, activates all neurons). Calcium influx was analyzed by the mean fluorescence measured with the LAS X Software (Leica Microsystems, Mannheim, Germany). Each plate consisted of a pool of DRG obtained of 3 animals, and each group consisted of 3 plates per group (*N* = 27).

### Stomach toxicity assay

The myeloperoxidase (MPO) activity was used to determine stomach toxicity, as this is a reliable marker for non-steroidal anti-inflammatory drug induced lesions [44]. A positive control used was daily i.p. treatment with indomethacin (2.5 mg/kg) over 7 days [48]. After 7 h or 7 days, stomach samples were collected in 50 mM K_2_HPO_4_ buffer (pH 6.0) containing 0.5% hexadecyl trimethylammonium bromide (HTAB) and kept at −80 °C until use. Frozen samples were homogenized using a tissue turrax (Tissue-Tearor 985,370, BioSpec Products, Bartlesville, OK, USA), centrifuged (2 min, 14.000 rpm, 4 °C), and the resulting supernatant was assayed using a spectrophotometer (Multiskan GO Microplate Spectrophotometer, Thermo Scientific, Vantaa, Finland) to determine MPO activity at 450 nm. The samples’ MPO activity was compared to a standard curve of neutrophils. Briefly, 15 µL of the sample was mixed with 200 µL of 50 mM phosphate buffer (pH 6.0) containing 0.167 mg/mL of O- dianisidinedihydrochloride and 0.015 hydrogen peroxide. Results were represented as MPO activity (number of neutrophils/mg of tissue). Each group consisted of 6 animals (*N* = 24).

### Liver and kidney toxicity assays

Elevated plasma ALT and AST levels are indicative of hepatic damage [40], as well as high levels of creatinine and urea in plasma are biomarkers of kidney damage [41]. For the ALT and AST tests, the positive control used was a once-oral treatment with acetaminophen (650 mg/kg), with plasma collected 24 h after treatment [42]. For the urea and creatinine assays, the positive control used was a once-oral treatment with diclofenac (200 mg/kg), with plasma collected 24 h after treatment [49]. Thus, after 7 h or 24 h, blood was harvested in microtubes containing 50 µL of the anticoagulant heparin (5000 IU/mL), centrifuged (200 × g, 10 min, 4 °C), and the plasma was separated. To determine enzymatic activities of AST and ALT as indicators of hepatotoxicity compared to acetaminophen, and to ascertain urea and creatinine levels as indicators of nephrotoxicity compared to diclofenac, plasma samples were processed according to the manufacturer’s instructions (Labtest Diagnóstico S.A, Lagoa Santa, Brazil). Results were presented as U/mL (ALT and AST) or mg/dL (urea and creatinine) of plasma. Each group consisted of 6 animals (*N* = 30).

### Data analysis

The sample size was determined using the G*Power software (3.1.9.7) and varied accordingly to the test, which was described in Sect. "Materials and methods". Results are presented at means ± SEM of measurements made on six mice in each group per experiment. Each experiment was conducted twice. Table 1 summarizes the statistical analyzes and n for all figures in this manuscript. Data were analyzed using the software GraphPad Prism 9.0. Data normality was verified by the Shapiro–Wilk test. In normal data, one-way or two-way ANOVA tests (depending on the variables) followed by Tukey’s post hoc or Sidak’s post hoc were used. For data without normal distribution, the non-parametric Kruskal–Wallis test followed by Dunn’s post hoc was used. For all analyzes, differences were considered significant for *P* < 0.05.

**Table 1:** Statistical information of the results

Result	Group	N	Statistical test	Post-test	F value	H value	*P* value
Figure 2A (Mechanical hyperalgesia)	Saline KO_2_ + Vehicle KO_2_ + PDX 1 ng KO_2_ + PDX 3 ng KO_2_ + PDX 10 ng	6 6 6 6 6	Two-way ANOVA	Tukey	Time × Dose: F (16, 100) = 2.528 Time: F (4, 100) = 3.323 Dose: F (4, 25) = 96.48 Subject: F (25, 100) = 7.825	–	*P* = 0.0027 *P* = 0.0134 *P* < 0.0001 *P* < 0.0001
Figure 2B (Thermal hyperalgesia – hot plate)	Saline KO_2_ + Vehicle KO_2_ + PDX 1 ng KO_2_ + PDX 3 ng KO_2_ + PDX 10 ng	6 6 6 6 6	Two-way ANOVA	Tukey	Time × Dose: F (20, 125) = 4.837 Time: F (5, 125) = 65.02 Dose: F (4, 25) = 84.27 Subject: F (25, 125) = 1.374	–	*P* < 0.0001 *P* < 0.0001 *P* < 0.0001 *P* = 0.1300
Figure 2C (Mechanical allodynia)	Saline KO_2_ + Vehicle KO_2_ + PDX 10 ng	6 6 6	Two-way ANOVA	Tukey	Time × Dose: F (10, 75) = 81.09 Time: F (5, 75) = 172.9 Dose: F (2, 15) = 671.8 Subject: F (15, 75) = 1.916	–	*P* < 0.0001 *P* < 0.0001 *P* < 0.0001 *P* = 0.0345
Figure 2D (Thermal hyperalgesia – Hargreaves)	Saline KO_2_ + Vehicle KO_2_ + PDX 10 ng	6 6 6	Two-way ANOVA	Tukey	Time × Dose: F (10, 75) = 25.25 Time: F (5, 75) = 95.90 Dose: F (2, 15) = 195.5 Subject: F (15, 75) = 3.396	–	*P* < 0.0001 *P* < 0.0001 *P* < 0.0001 *P* = 0.0002
Figure 3A (Writings)	Saline KO_2_ + Vehicle KO_2_ + PDX 10 ng	6 6 6	One-way ANOVA	Tukey	F (2, 15) = 25.09	–	*P* < 0.0001
Figure 3B (Flinches)	Saline KO_2_ + Vehicle KO_2_ + PDX 10 ng	10 10 10	One-way ANOVA	Tukey	F (2, 27) = 47.91	–	*P* < 0.0001
Figure 3C (Licking)	Saline KO_2_ + Vehicle KO_2_ + PDX 10 ng	10 10 10	One-way ANOVA	Tukey	F (2, 27) = 26.93	–	*P* < 0.0001
Figure 3D (SWB)	Saline KO_2_ + Vehicle KO_2_ + PDX 10 ng	6 6 6	Two-way ANOVA	Tukey	Time x Dose: F (8, 60) = 5.825 Time: F (4, 60) = 11.29 Dose: F (2, 15) = 77.85 Subject: F (15, 60) = 0.795	–	*P* < 0.0001 *P* < 0.0001 *P* < 0.0001 *P* = 0.6772
Figure 4A (Score of histological parameters)	Saline KO_2_ + Vehicle KO_2_ + PDX 10 ng	4 4 4	Kruskal–Wallis	Dunn’s	–	12.06	*P* = 0.0002
Figure 4B (Leukocyte infiltrate)	Saline KO_2_ + Vehicle KO_2_ + PDX 10 ng	4 4 4	One-way ANOVA	Tukey	F (2, 9) = 51.12	–	*P* < 0.0001
Figure 4I (Leukocyte infiltrate in LysM-eGFP)	Saline KO_2_ + Vehicle KO_2_ + PDX 10 ng	10 10 10	One-way ANOVA	Tukey	F (2, 27) = 144.7	–	*P* < 0.0001
Figure 5A (TNF-α)	Saline KO_2_ + Vehicle KO_2_ + PDX 10 ng	10 10 10	One-way ANOVA	Tukey	F (2, 27) = 9.925	–	*P* = 0.0006
Figure 5B (IL-1β)	Saline KO_2_ + Vehicle KO_2_ + PDX 10 ng	10 10 10	One-way ANOVA	Tukey	F (2, 27) = 57.18	–	*P* < 0.0001
Figure 6A (GSH)	Saline KO_2_ + Vehicle KO_2_ + PDX 10 ng	10 10 10	One-way ANOVA	Tukey	F (2, 27) = 16.57	–	*P* < 0.0001
Figure 6B (Catalase)	Saline KO_2_ + Vehicle KO_2_ + PDX 10 ng	10 10 10	One-way ANOVA	Tukey	F (2, 27) = 12.28	–	*P* = 0.0002
Figure 6C (FRAP)	Saline KO_2_ + Vehicle KO_2_ + PDX 10 ng	10 10 10	One-way ANOVA	Tukey	F (2, 27) = 22.49	–	*P* < 0.0001
Figure 6D (ABTS)	Saline KO_2_ + Vehicle KO_2_ + PDX 10 ng	10 10 10	One-way ANOVA	Tukey	F (2, 27) = 9.790	–	*P* = 0.0006
Figure 6E (NBT)	Saline KO_2_ + Vehicle KO_2_ + PDX 10 ng	10 10 10	One-way ANOVA	Tukey	F (2, 27) = 18.68	–	*P* < 0.0001
Figure 7A (TRPV1)	Saline KO_2_ + Vehicle KO_2_ + PDX 10 ng	10 10 10	One-way ANOVA	Tukey	F (2, 27) = 20.64	–	*P* < 0.0001
Figure 7B (pNFκB)	Saline KO_2_ + Vehicle KO_2_ + PDX 10 ng	10 10 10	One-way ANOVA	Tukey	F (2, 27) = 8.69	–	*P* = 0.0012
Figure 7C (TRPV1-pNFκB)	Saline KO_2_ + Vehicle KO_2_ + PDX 10 ng	10 10 10	One-way ANOVA	Tukey	F (2, 27) = 15.23	–	*P* < 0.0001
Figure 8C (Fluo-4AM fluorescence per time)	Saline KO_2_ + Vehicle KO_2_ + PDX 10 ng	3 3 3	Two-way ANOVA	Tukey	Time × Dose: F (2, 6) = 0.3780 Time: F (1, 6) = 11.95 Dose: F (2, 6) = 13.07 Subject: F (6, 6) = 2.316	–	*P* = 0.7005 *P* = 0.0135 *P* = 0.0065 *P* = 0.1651
Figure 9A (MPO activity)	Saline KO_2_ + Vehicle KO_2_ + PDX 10 ng Indomethacin (2.5 mg/kg)	6 6 6 6	One-way ANOVA	Tukey	F (3, 20) = 7.647	–	*P* = 0.0013
Figure 9B (ALT)	Saline KO_2_ + Vehicle KO_2_ + PDX 10 ng Acetaminophen (650 mg/kg)	6 6 6 6	One-way ANOVA	Tukey	F (3, 20) = 7.493	–	*P* = 0.0015
Figure 9C (AST)	Saline KO_2_ + Vehicle KO_2_ + PDX 10 ng Acetaminophen (650 mg/kg)	6 6 6 6	One-way ANOVA	Tukey	F (3, 20) = 12.95	–	*P* < 0.0001
Figure 9D (Urea)	Saline KO_2_ + Vehicle KO_2_ + PDX 10 ng Diclofenac (200 mg/kg)	6 6 6 6	One-way ANOVA	Tukey	F (3, 20) = 7.647	–	*P* = 0.0013
Figure 9E (Creatinine)	Saline KO_2_ + Vehicle KO_2_ + PDX 10 ng Diclofenac (2.5 mg/kg)	6 6 6 6	One-way ANOVA	Tukey	F (3, 20) = 7.647	–	*P* = 0.0013
Figure 10A (Mechanical hyperalgesia)	Saline PDX 10 ng	6 6	Two-way ANOVA	Sidak	Time × Dose: F (4, 40) = 0.9939 Time: F (4, 40) = 1.320 Dose: F (1, 10) = 0.3475 Subject: F (10, 40) = 1.993	–	*P* = 0.4221 *P* = 0.2791 *P* = 0.5686 *P* = 0.0602
Figure 10B (Thermal hyperalgesia–hot plate)	Saline PDX 10 ng	6 6	Two-way ANOVA	Sidak	Time × Dose: F (5, 50) = 0.4244 Time: F (5, 50) = 1.954 Dose: F (1, 10) = 0.001097 Subject: F (10, 50) = 2.407	–	*P* = 0.8295 *P* = 0.1019 *P* = 0.9742 *P* = 0.0201
Figure 10C (Thermal hyperalgesia–Hargreaves)	Saline PDX 10 ng	6 6	Two-way ANOVA	Sidak	Time × Dose: F (4, 40) = 0.8147 Time: F (4, 40) = 1.013 Dose: F (1, 10) = 0.03630 Subject: F (10, 40) = 2.182	–	*P* = 0.5234 *P* = 0.4123 *P* = 0.8527 *P* = 0.0396
Figure 10D (Rota rod)	Saline PDX 10 ng	6 6	Two-way ANOVA	Sidak	Time × Dose: F (5, 50) = 0.5680 Time: F (5, 50) = 0.5680 Dose: F (1, 10) = 4.175 Subject: F (10, 50) = 0.6802	–	*P* = 0.7240 *P* = 0.7240 *P* = 0.0683 *P* = 0.7374

SWB: static weight bearing; TNF-α, Tumor Necrosis Factor alpha; IL-1β, Interleukin-1 beta; GSH, Reduced Glutathione; FRAP, Ferric Reducing Antioxidant Power; ABTS, 2,2′-azino-bis(3-ethylbenzothiazoline-6-sulfonic acid; NBT, Nitroblue Tetrazolium; TRPV1, Transient Receptor Potential Vanilloid 1, pNFκB, phosphorylated Nuclear Factor kappa B; MPO, myeloperoxidase; ALT, Alanine Aminotransferase; AST, Aspartate Aminotransferase. The n for each group in the Fig. 8C is of 3 plates, and each plate was a pool of 3 mice. The n reported for all other figures is of number of mice per group

## Results

### PDX decreases KO_2_-induced mechanical hyperalgesia, thermal hyperalgesia, and mechanical allodynia

**Fig. 2:**
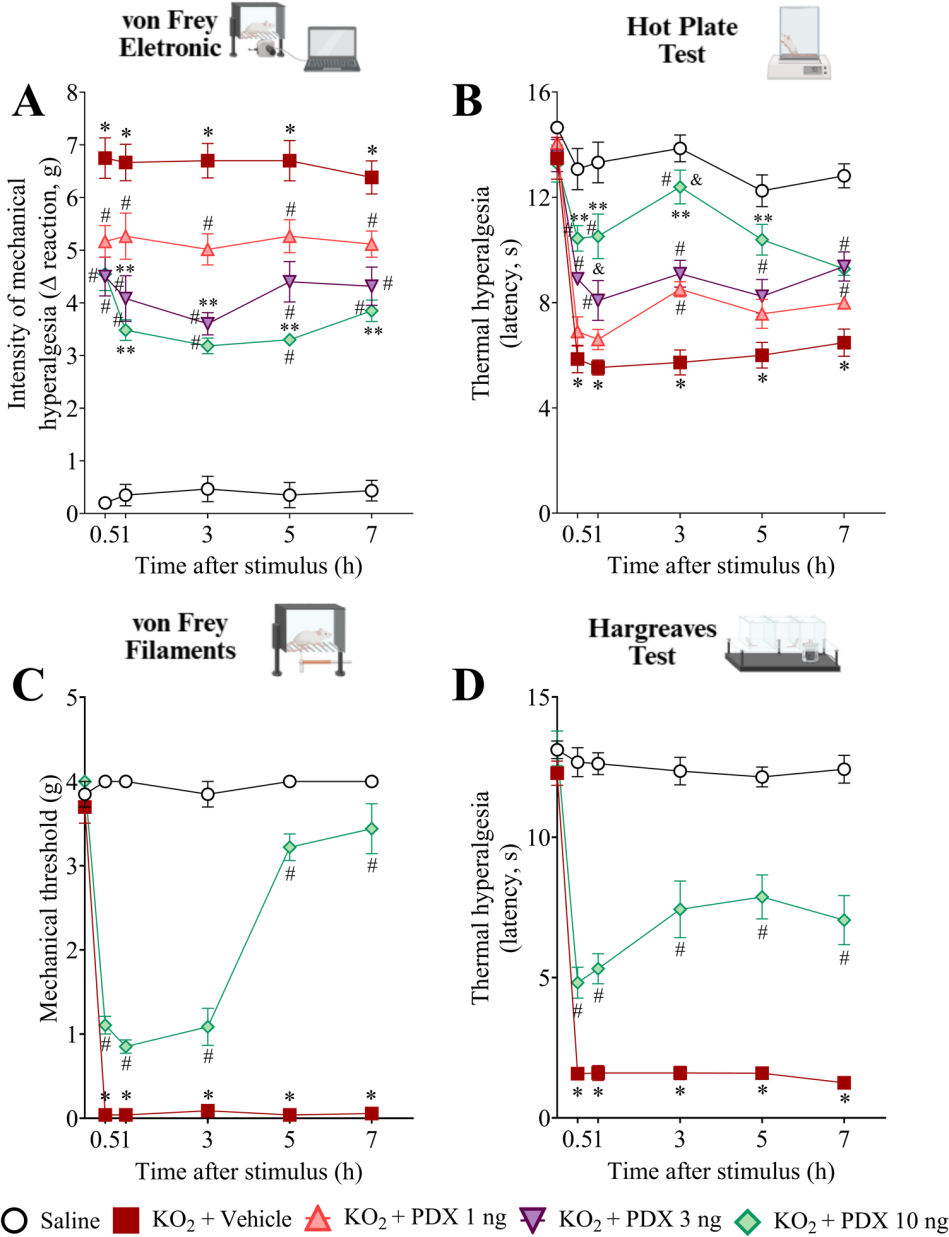
PDX decreases KO_2_-induced mechanical hyperalgesia, thermal hyperalgesia and mechanical allodynia. Mechanical hyperalgesia intensity (**A**), thermal hyperalgesia (**B**, **D**) and mechanical allodynia (**C**) was evaluated 0 (baseline), 0.5, 1, 3, 5 and 7 h after KO_2_ (30 µg/20 µL/paw, i.pl.) or vehicle injection (sterile saline, NaCl 0.9%, 20 µL/paw, i.pl.), using an electronic von Frey, hot plate apparatus, Hargreaves apparatus and von Frey manual filaments, respectively. Results are presented as Δ mean n ± SEM; n = 6 mice per group per experiment (*P < 0.05 vs saline group; #*P* < 0.05 vs vehicle group; **P < 0.05 vs 1 ng PDX; & *P* < 0.05 vs 3 ng PDX [two-way ANOVA followed by Tukey’s post hoc test])

Initially, we evaluated the efficacy of PDX treatment in KO_2_-induced mechanical and thermal hyperalgesia. PDX (1, 3, and 10 ng, 100 µL, i.p.) significantly reduced the mechanical hyperalgesia at all times of analysis (Fig. 2A). In the thermal hyperalgesia analyzes, determined using the hot plate test (Fig. 2B), the doses 3 and 10 ng of PDX (100 µL, i.p.), reduced this parameter from the 0.5 h until the 7 h after the stimulus. The 1 ng dose showed antinociceptive potential only in the 3rd h. Furthermore, when statistically compared with the other doses of 1 and 3 ng, 10 ng PDX (100 µL, i.p.) demonstrated a more efficient antinociceptive performance, therefore, it was selected for the subsequent experiments.

Subsequently, PDX (10 ng, 100 µL, i.p.) decreased mechanical allodynia (Fig. 2C), as well as reduced thermal hyperalgesia assessed by Hargreaves Test (Fig. 2D), in all measurement times. Then, these results demonstrated that PDX attenuated evoked pain parameters.

### PDX reduces KO_2_-induced overt pain-like behaviors and the unbalanced body weight distribution between the hind paws

**Fig. 3:**
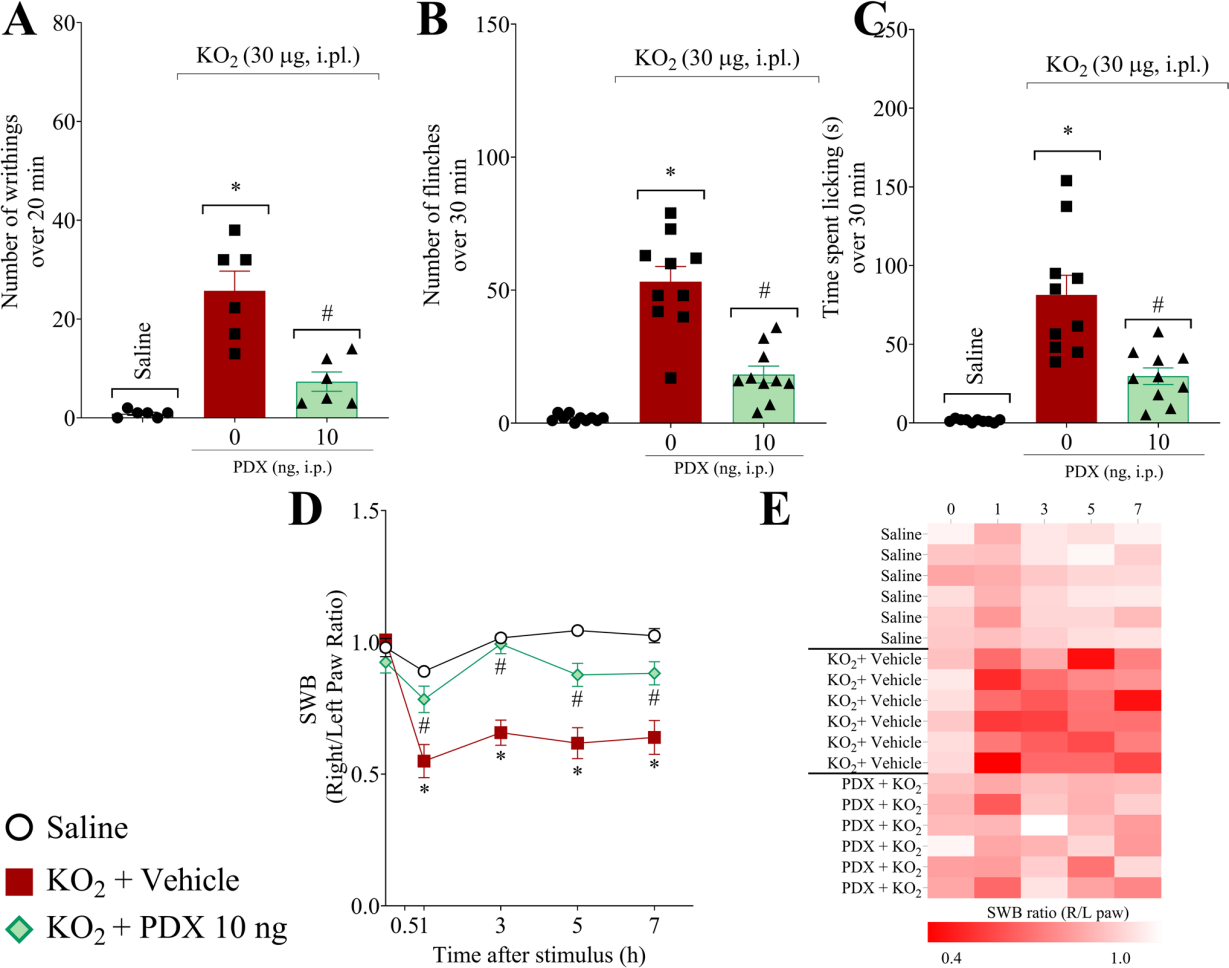
PDX reduces KO_2_-induced overt pain-like behaviors and unbalanced weight distribution in the hind paws. The number of abdominal writhings over 20 min. **A** after KO_2_ (1 mg/100 µL/peritoneal cavity, i.p.) or vehicle injection (sterile saline, NaCl 0.9%, 100 µL/peritoneal cavity, i.p.). The number of paw flinches (**B**) and time spent licking the paw (**C**) were determined over 30 min after KO_2_ (30 µg/20 µL/paw, i.pl.) or vehicle injection (sterile saline, NaCl 0.9%, 20 µL/paw, i.pl.). Results are presented as total number of writhings (over 20 min) (mean n ± SEM; n = 6 mice per group per experiment), flinches and time spent licking the paw (over 30 min) (mean n ± SEM; n = 10 mice per group per experiment) (*P < 0.05 vs saline group; #*P* < 0.05 vs vehicle group). SWB was used as a nonreflexive method of pain measurement (**D**), and this parameter was evaluated 1, 3, 5, and 7 h after stimulus. The heat map shows the right/left hind paw ratio of each mouse (**E**). Results are presented as weight ratio of left/right hind paw; n = 6 mice per group per experiment (*P < 0.05 vs saline group; #*P* < 0.05 vs vehicle group [one-way ANOVA followed by Tukey’s post hoc test (**A–C**) or two-way ANOVA followed by Tukey’s post hoc test (**B**)])

The efficacy of PDX in KO_2_-induced non-evoked pain was evaluated (Fig. 3). Mice were treated once with PDX (10 ng, 100 µL, i.p.) or vehicle (0.7% ethanol in sterile saline, NaCl 0.9%, 100 µL, i.p.), 1 h before receiving KO_2_ or vehicle injection. The abdominal writhings were determined over 20 min after i.p. KO_2_ injection (1 mg, 100 µL, i.p.) (Fig. 3A). PDX (10 ng, 100 µL, i.p.) significantly reduces the KO_2_-induced number of abdominal writhings (Fig. 3A). The paw flinches (Fig. 3B) and time spent licking the paw (Fig. 3C) were determined over 30 min after i.pl. KO_2_ injection (30 µg, 20 µL, i.pl.). PDX (10 ng, 100 µL, i.p.) reduced the KO_2_-induced number of paw flinches (Fig. 3B) and the time spent licking the paw (Fig. 3C).

In addition, we assessed the weight distribution between the hind paws by SWB apparatus, a nonreflexive method in which the mouse distributes the weight between hind paws without interference of the experimenter. PDX (10 ng) increased the right/left paw ratio, closer to the ratio value of 1 (Fig. 3D), which reflects the reduction in unbalanced weight distribution on the hind paw. The heat map shows the right/left hind paw ratio of each mouse (Fig. 3E). Then, these data reinforce the PDX antinociceptive activity.

### PDX attenuates KO_2_-induced leukocyte migration to the paw

**Fig. 4:**
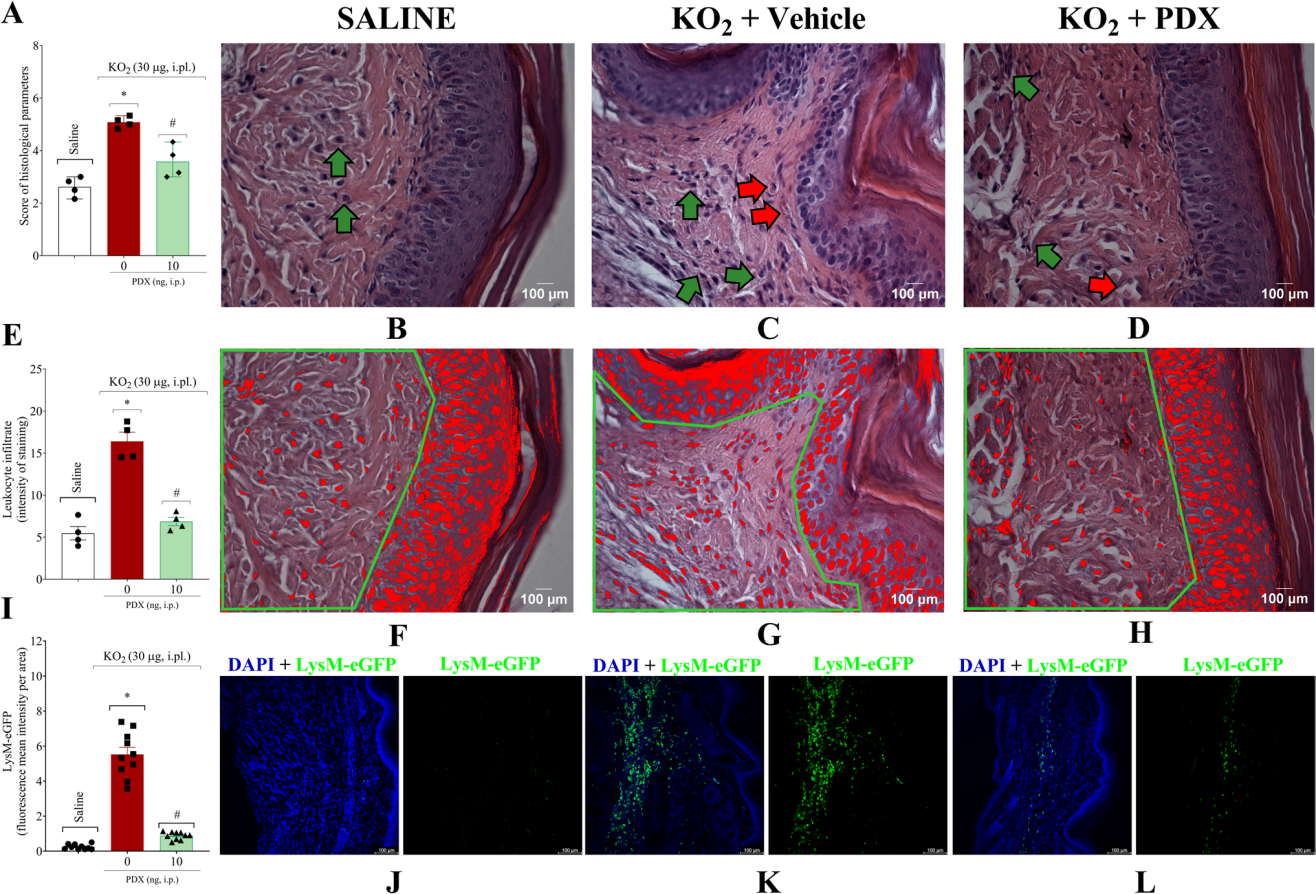
PDX attenuates KO_2_-induced leukocyte migration to the paw. Seven h after KO_2_ (30 µg/20 µL/paw, i.pl.) or vehicle injection (sterile saline, NaCl 0.9%, 20 µL/paw, i.pl.), the Swiss mice hind paw skin (**A–H**) was dissected for histopathological analysis by H&E staining using a light microscope (original magnification 40x). In (**B**–**D**) showing the leukocyte infiltrate (green arrow) and angiogenesis (red arrow). The analysis was performed using a clinical score (**A**) where neovascularization, tissue integrity and inflammatory infiltrate (**A**–**D**) were evaluated and the results were expressed as median + range (n = 4 mice per group). The inflammatory infiltrate was expressed for intensity of staining per area (highlighted area in green (**E–H**)) using the Image J software of each group with 4 mice. In addition, the plantar tissue of LysM-eGFP C57BL/6 mice (**I**–**L**) was dissected 7 h after stimulus for the determination of fluorescence intensity using a confocal microscope. Intensity of fluorescence is represented in (**I**) and representative images are shown in (**J**–**L**) (N = 10 mice per group). *P < 0.05 vs saline group; #*P* < 0.05 vs vehicle group, non-parametric Kruskal–Wallis test was used, followed by Dunn’s test, median + range (**A**); One-way ANOVA followed by Tukey’s post-test, mean ± SEM (**B**, **I**)

The hind paw skin was collected 7 h after KO_2_ or vehicle injection to evaluate the PDX effect on leukocyte migration (Fig. 4). Firstly, we assessed the leukocyte recruitment in plantar tissue of swiss mice using H&E staining (Fig. 4A–H), focusing on the dermal region in the hind paw skin. In the clinical score images (Fig. 4A–D), the green arrow indicates the leukocyte infiltrate, and the red arrow the angiogenesis process. PDX presents a better score compared to the vehicle group, with reduced leukocyte infiltrate, angiogenesis, and tissue damage. In addition, PDX (10 ng, 100 µL, i.p.) reduced the leukocyte migration in the demarcated area of the dermis (Fig. 4E–H), which was confirmed by statistical analysis (Fig. 4B).

Moreover, we used LysM-eGFP mice that have the enhanced green fluorescent protein (eGFP) inserted into the promoter region of Lysozyme M (LysM), which allowed a specific analysis of PDX effect on the recruitment of neutrophils and macrophages, acute inflammation characteristic cells (Fig. 4I–L). PDX (10 ng, 100 µL, i.p.) reduced the recruitment of neutrophils and macrophages into the dermis (Fig. 4I–J). These results evidenced the PDX anti-inflammatory activity.

It is noteworthy that there is some apparent discrepancy between the H&E and LysM-eGFP data because the basal detection of cellular infiltrate was higher in the saline group applying the H&E method (Fig. 4A and E) than LysM-eGFP detection (Fig. 4I). A likely explanation is that in the case of LysM-eGFP, lysozyme M expression in tissue resident cells is low while in recruited macrophages and neutrophils it is high [50–52]. Therefore, LysM-eGFP fluorescent detection data would more accurately represent the recruited cells while H&E analyzes would in addition include tissue resident cells.

### PDX reduces KO_2_-induced pro-inflammatory cytokines production

**Fig. 5:**
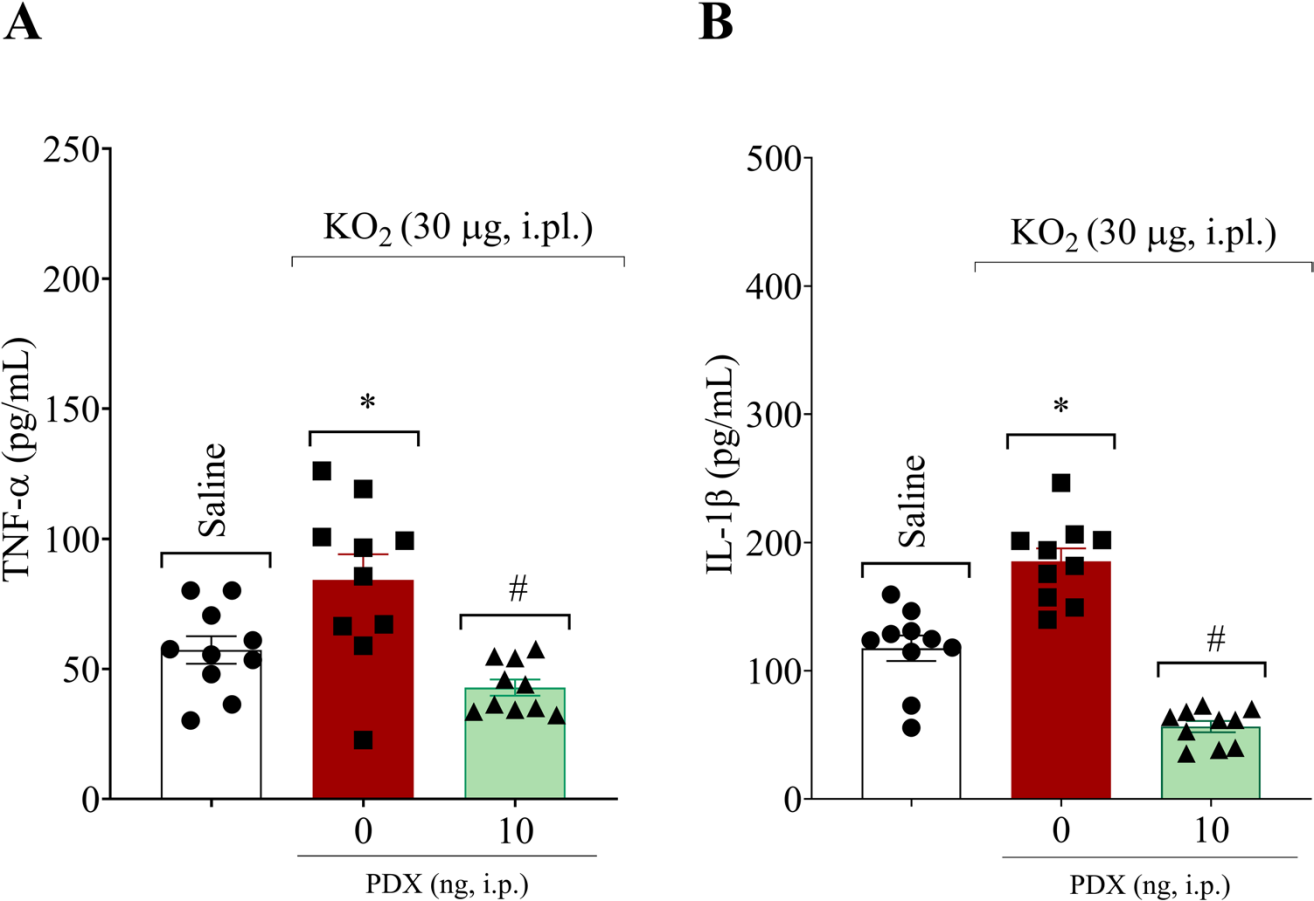
PDX reduces KO_2_-induced TNF-α and IL-1β production. The proinflammatory cytokines TNF-α (**A**) and IL-1β (**B**) levels were assayed by ELISA. Hind paw skin was dissected 2 h after KO_2_ (30 µg/20 µL/paw, i.pl.) or vehicle injection (sterile saline, NaCl 0.9%, 20 µL/paw, i.pl.), processed using a surgical scalpel blade and cultivated in culture medium for 2 h (a total of 4 h after KO_2_ injection). Results are expressed as mean ± SEM; n = 10 mice per group per experiment (*P < 0.05 vs saline group; #*P* < 0.05 vs vehicle group; [one-way ANOVA followed by Tukey’s post hoc test])

The hind paw skin was dissected 2 h after the KO_2_ or vehicle injection. Sequentially, samples were fragmented individually into four small pieces using a surgical scalpel blade by performing one vertical and one horizontal cut across the center of the specimen. The fragments were cultivated in a culture medium for an additional 2 h (a total of 4 h after KO_2_ injection). Next, the culture supernatant was collected for cytokine quantification to evaluate the PDX effect on the production of the proinflammatory cytokines TNF-α and IL-1β (Fig. 5). PDX (10 ng, 100 µL, i.p.) efficiently decreased the KO_2_-triggered production of TNF-α (Fig. 5A) and IL-1β (Fig. 5B), demonstrating an important anti-inflammatory effect of this SPM.

### PDX increases endogenous antioxidant systems, normalizes total antioxidant capacity, and attenuates superoxide anion production

**Fig. 6:**
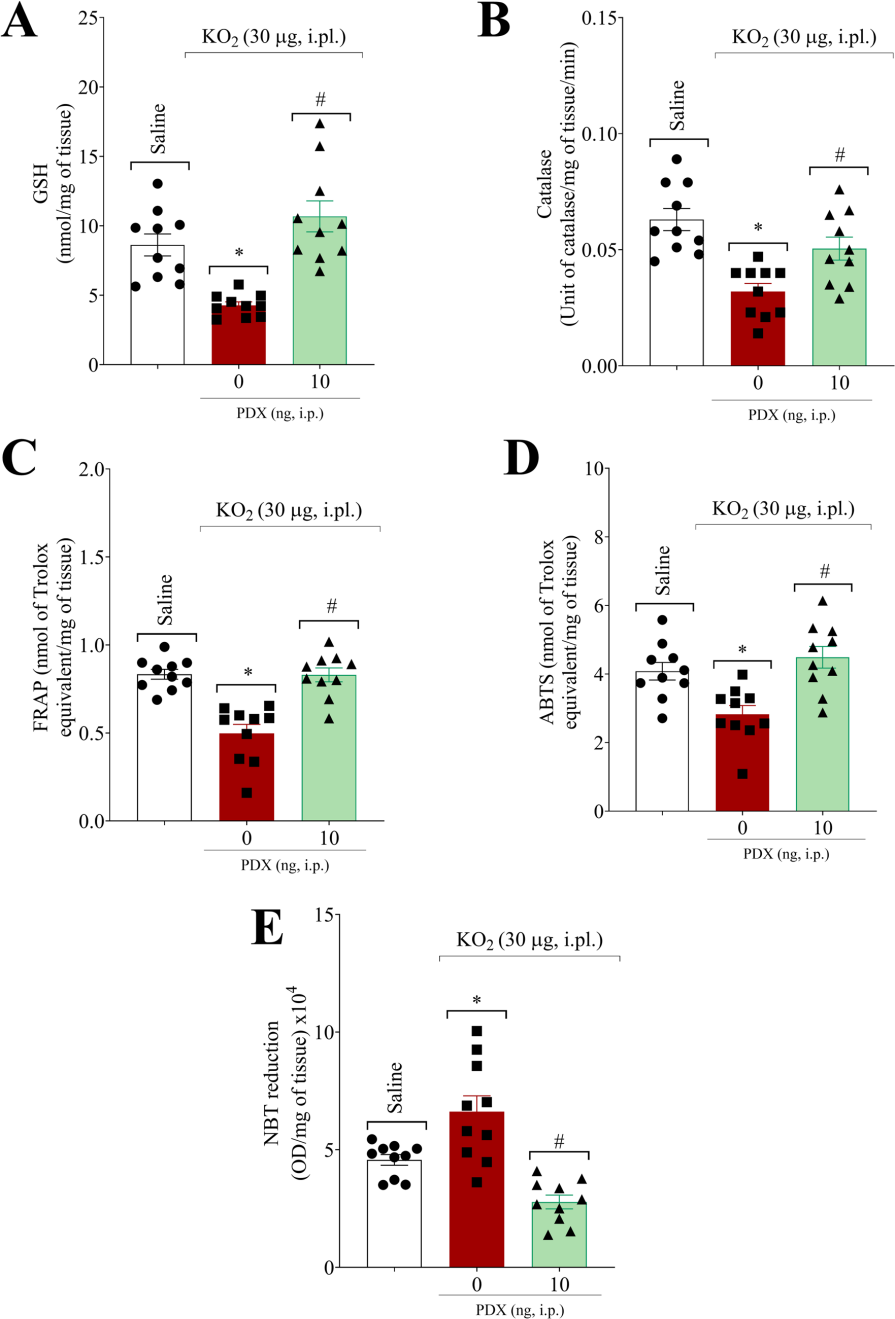
PDX increases endogenous antioxidant systems, normalizes total antioxidant capacity, and reduces superoxide anion production. Hind paw was dissected 1 h or 3 h after KO_2_ (30 µg/20 µL/paw, i.pl.) or vehicle injection (sterile saline, NaCl 0.9%, 20 µL/paw, i.pl.) for determination of GSH content (**A**) and Catalase activity (**B**), total antioxidant capacity (**C**, FRAP assay, **E**, ABTS assay), and superoxide anion production (**E**) using colorimetric assays. Results are expressed as mean ± SEM; n = 10 mice per group per experiment. (*P < 0.05 vs saline group; #*P* < 0.05 vs vehicle group; [one-way ANOVA followed by Tukey’s post hoc test])

Considering the importance of ROS for the KO_2_-triggered inflammatory pain model, we assessed the PDX effect on oxidative stress (Fig. 6). PDX (10 ng, 100 µL, i.p.) improved the endogenous antioxidant systems, increasing the GSH content (Fig. 6A) and the catalase activity (Fig. 6B). Furthermore, PDX (10 ng, 100 µL, i.p.) restored the total antioxidant capacity as observed by the normalization of the capacity to reduce ferric ions (FRAP assay, Fig. 6C) and scavenger ability (ABTS assay, Fig. 6D). In addition, PDX attenuated superoxide anion production (NBT assay, Fig. 6E), demonstrating efficient potential to induce ROS neutralization.

### PDX decreases KO_2_-induced TRPV1 and NF-κB activation in DRG neurons

**Fig. 7:**
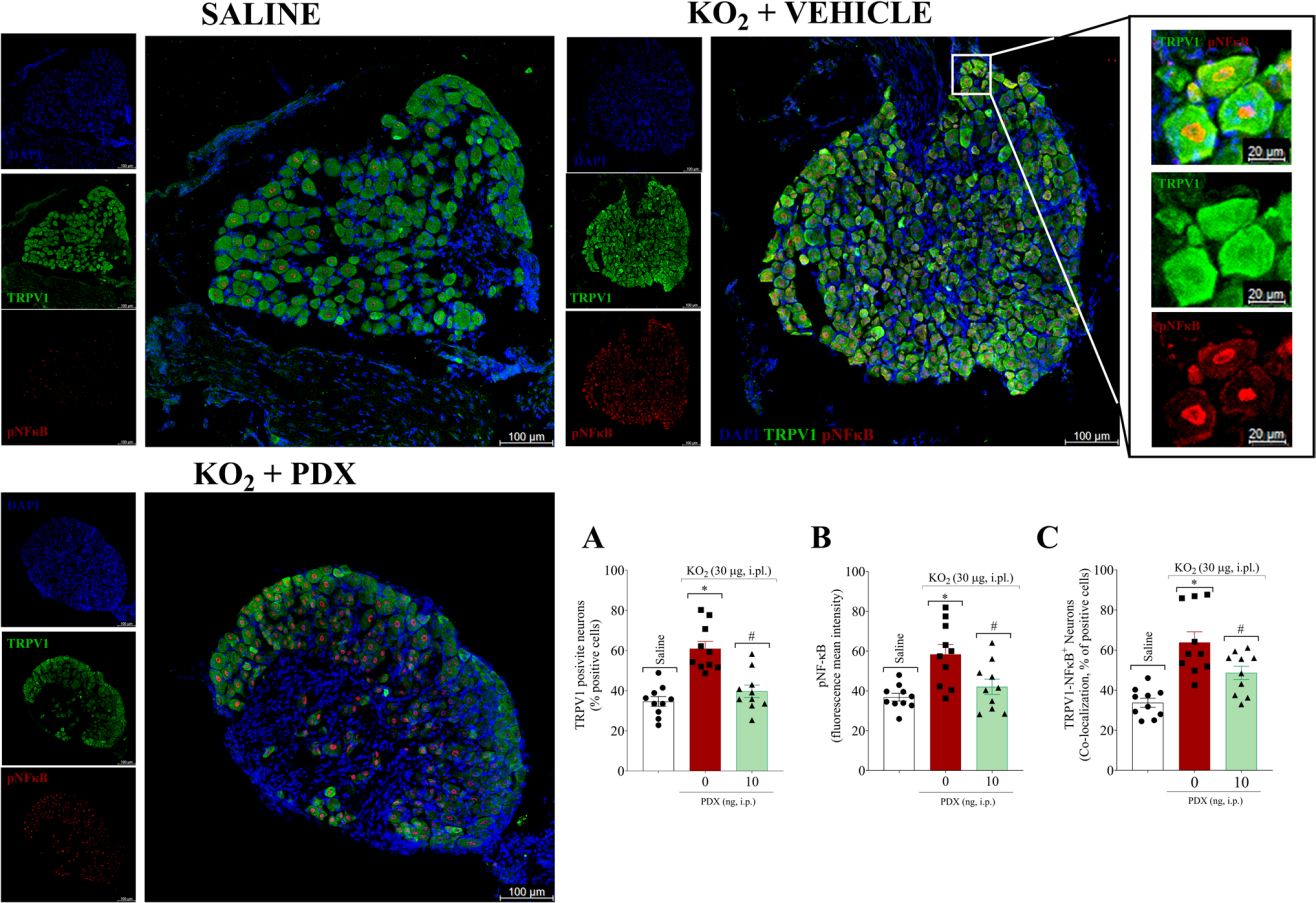
PDX decreases KO_2_-induced TRPV1 and NF-κB activation in DRG neurons. Five h after KO_2_ (30 µg/20 µL/paw, i.pl.) or vehicle injection (sterile saline, NaCl 0.9%, 20 µL/paw, i.pl.), DRG samples (L4-L6) were dissected for immunofluorescent TRPV1 and p65 p-NF-κB staining. The graphs show the percent of (**A**) TRPV1 positive cells, (**B**) p-NFκB positive cells, and (**C**) TRPV1 positive cells co-stained with p-NFκB. Representative images shows nuclear staining with DAPI (blue), TRPV1 positive cells (green), p-NFκB positive cells (red), TRPV1 + p-NFκB positive cells (green + red) in DRGs group (20 × magnification with 0.75 × zoom). The nuclear localization of p-NF-kB in TRPV1^+^ cells can be better observed in the magnification performed on the representative of the KO_2_ (Zoom 2×). The Results are expressed as mean ± SEM; n = 10 mice per group per experiment) (*P < 0.05 vs saline group; #*P* < 0.05 vs vehicle group; [one-way ANOVA followed by Tukey’s post hoc test])

Considering the importance of identifying a pharmacological target in nociceptive neurons for the management of inflammatory pain, our next step was to evaluate the PDX effect on TRPV1 and neuronal activation (Fig. 7). In terms of neuronal function, the immunofluorescence data demonstrated that KO_2_ injection increased the percentage of TRPV1^+^ neurons, p-NFκB^+^ neurons, and TPRV1^+^ neurons co-stained with p-NFκB, and PDX (10 ng, 100 µL, i.p.) reversed this response (Fig. 7A–C). PDX reduces TRPV1 (Fig. 7A) and pNF-κB (Fig. 7B) staining in DRG, which reflects the inhibition of neuronal activation. Figure 7 shows representative images of groups. Furthermore, PDX decreased the co-stained TRPV1 with p-NF-κB p65 (Fig. 7C), demonstrating its ability to inhibit the activation of TRPV1^+^ neurons since p-NF-κB was applied as a surrogate marker of cellular activation. The nuclear localization of p-NF-kB in TRPV1^+^ cells can be better observed in the magnification performed on the representative of the KO_2_ group.

These data further demonstrate that TRPV1^+^ neurons are activated in KO_2_ inflammation and that PDX treatment reduces their activation, conferring an antinociceptive mechanism of this drug at the neuronal level.

### PDX reduces KO_2_-induced TRPV1 activation on DRG neurons

**Fig. 8:**
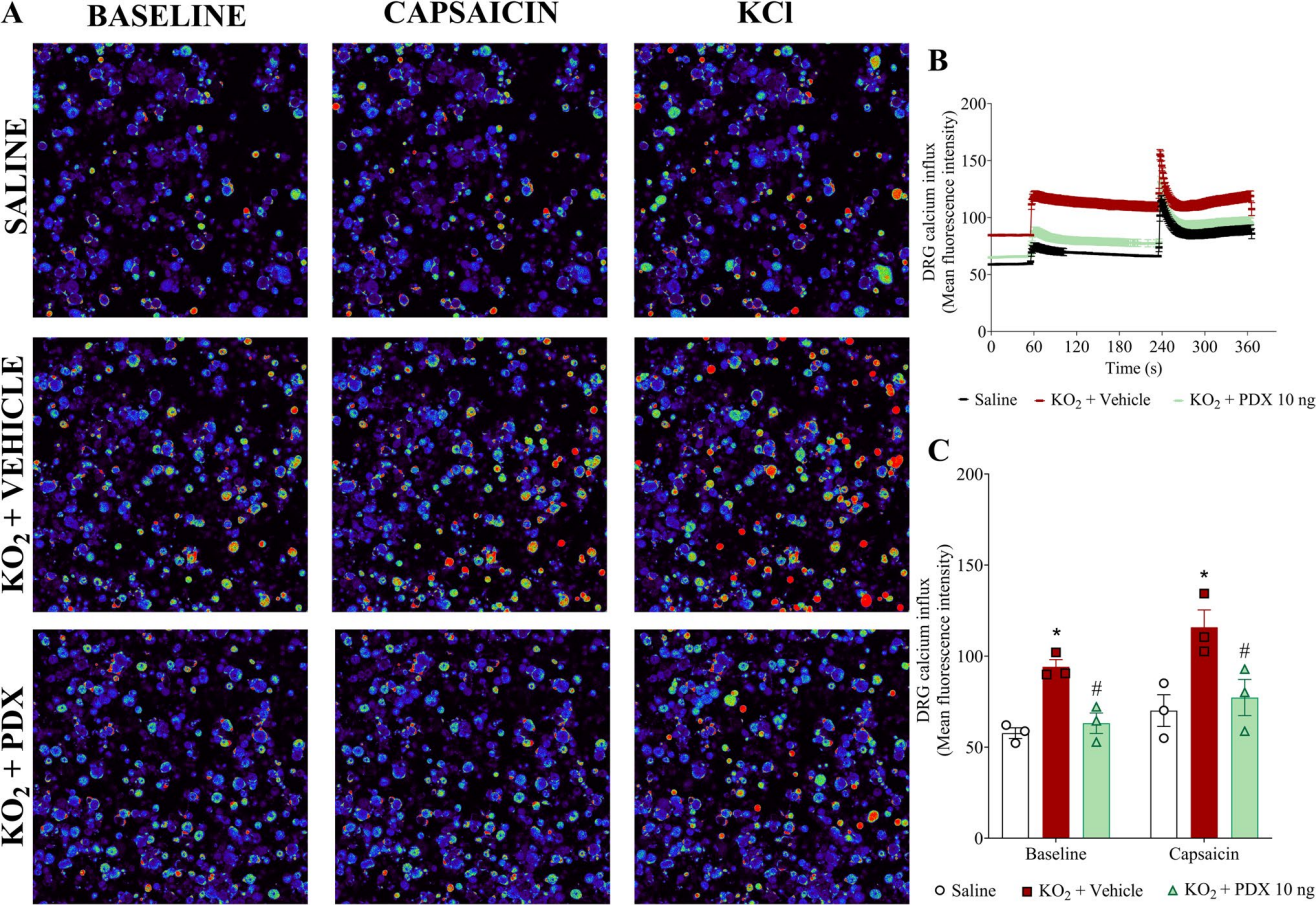
PDX reduces KO_2_-induced activation of TRPV1^+^ DRG neurons. Five h after KO_2_ (30 µg/20 µL/paw, i.pl.) or vehicle injection (sterile saline, NaCl 0.9%, 20 µL/paw, i.pl.), DRG samples (L4-L6) were dissected for calcium imaging using Fluo-4AM probe. The fluorescence intensity traces of calcium-fluo-4 in representative DRG fields during the 6 min of recording in shown in panel (**A**). **B** displays the mean fluorescence intensity of calcium-fluo-4 at baseline (0-s mark) and that following the stimulus with capsaicin (a TRPV1 agonist, 120-s mark). Response to KCl (activates all neurons) begins at the 240-s mark. **C** shows representative fields of DRG neurons (baseline fluorescence, the fluorescence after capsaicin, and after KCl. Results are expressed as mean ± SEM, n = 3 DRG seeded plates (each plate is a neuronal culture pooled from 9 mice) per group. (*P < 0.05 vs saline group; #*P* < 0.05 vs vehicle group; [two-way ANOVA followed by Tukey’s post hoc test])

Considering the results of Fig. 7, our next step was to assess neuronal activation by quantifying calcium influx as measured by a fluorescent probe Fluo-4AM in DRG neurons. Therefore, we investigated whether DRG neurons from KO_2_-stimulated mice would present an increase in the baseline calcium levels and response to capsaicin (TRPV1 agonist) stimulation compared to saline-injected control mice, and the ability of PDX to modulate this response (Fig. 8). DRG neurons from vehicle-treated mice present a higher baseline level of calcium influx than saline or PDX-treated DRGs (Fig. 8A–C). These data suggest that PDX reduces the DRG neuron activation in KO_2_-induced inflammation because the increase in calcium influx is indicative of DRG neuron activity (Fig. 8A–C).

In addition, PDX reduced the responsiveness of DRG neurons to capsaicin (Fig. 8A–C). Thus, PDX inhibits KO_2_-induced activation of TRPV1^+^ DRG neurons. TRPV1 activation leads to calcium influx and it is an important channel in the sensitization process of nociceptive neurons. These results evidenced the PDX antinociceptive neuronal activity.

### Acute PDX treatment does not induce liver, kidney, or stomach damage

**Fig. 9:**
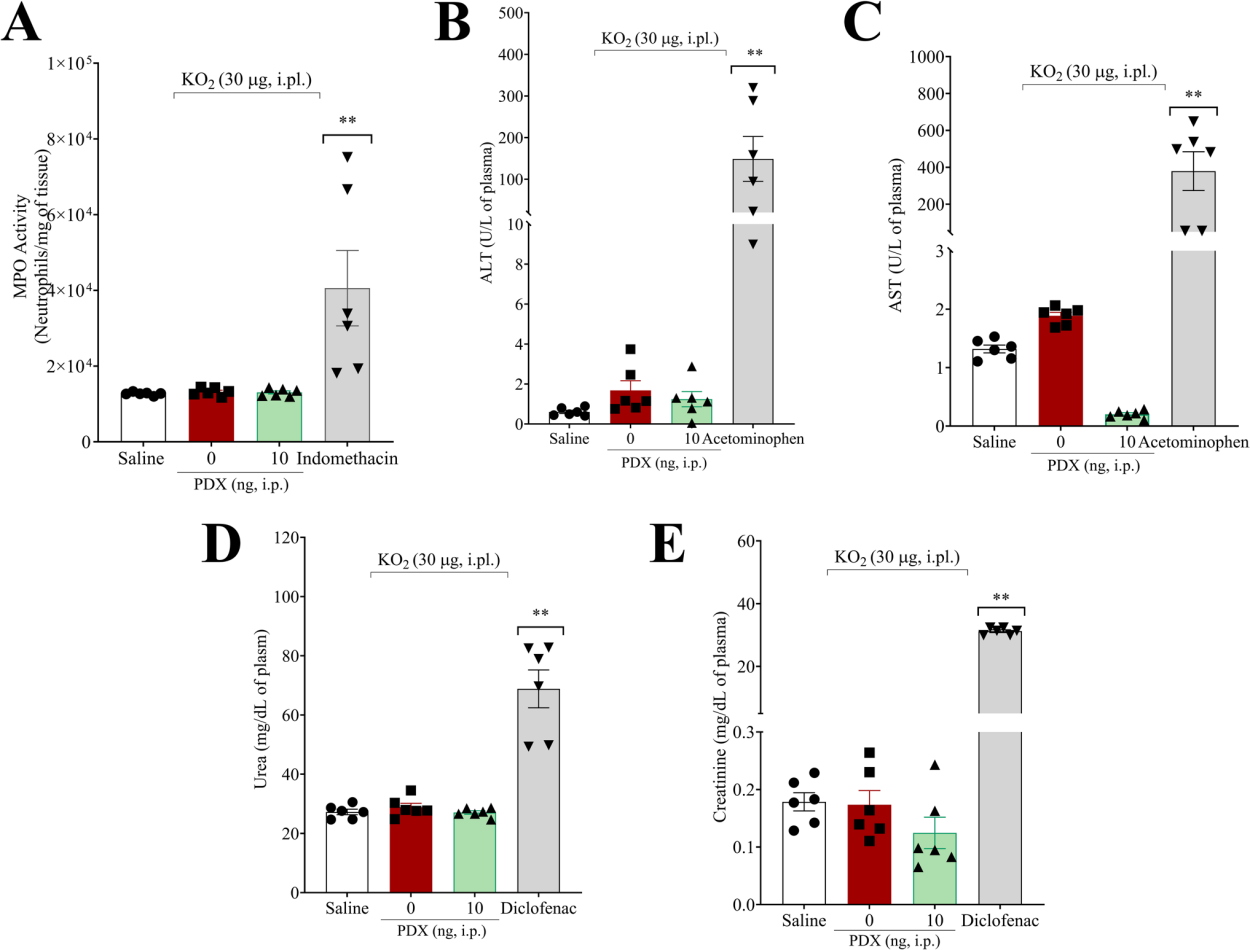
Acute PDX treatment does not induce liver, kidney or stomach damage. Mice were treated with PDX (10 ng, 100 µL, i.p.) 1 h before receiving the KO_2_ (30 µg/20 µL/paw, i.pl.) or vehicle injection (sterile saline, NaCl 0.9%, 20 µL/paw, i.pl.). There were also treatments with drugs that induce specific organ damage, which included acetaminophen (650 mg/kg, administered orally) for liver injury, diclofenac (200 mg/kg, administered orally) for kidney injury, and indomethacin (2.5 mg/kg, administered i.p.) for stomach injury. MPO activity was quantitated in samples of stomach (**A**), and the levels of ALT (**B**), AST (**C**), urea (**D**), and creatinine (**D**) were quantitated in the serum. Results are expressed as mean ± SEM; n = 6 mice per group per experiment. (**P < 0.05 vs saline group, vehicle group and PDX group [one-way ANOVA followed by Tukey’s post hoc test])

Mice received PDX (10 ng) 1 h before receiving the KO_2_ i.pl. injection. Seven h after the stimulus, plasma, and stomach samples were collected to evaluate whether the acute treatment with PDX would induce gastric, hepatic, or renal damage, which are common side effects of NSAIDs (Fig. 9). Toxicity was assessed through the plasmatic concentrations of AST, ALT, urea, creatinine, and stomach MPO activity as previously described [43, 53]. Mice were treated once with acetaminophen (650 mg/kg, orally) as a positive control for liver damage and had their plasma collected 24 h after treatment. Mice were treated once with diclofenac (200 mg/kg, orally) as a positive control for kidney damage, and plasma was collected 24 h after treatment. Finally, for positive control of stomach damage, the mice were treated daily with indomethacin over seven days (2.5 mg/kg, i.p.), with plasma collected on the 7th day after treatment. PDX (10 ng, 100 µL, i.p.) did not modify the MPO activity in the stomach (Fig. 9A), the plasmatic concentration of ALT (Fig. 9B), AST (Fig. 9C), urea (Fig. 9D), or creatinine (Fig. 9E) compared with positive controls. Therefore, our data demonstrate that a single PDX treatment does not induce detectable gastric, hepatic, or renal lesions/damage 7 h after its administration.

### PDX does not alter baseline mechanical sensitivity, heat sensitivity, and motor function in naïve mice

**Fig. 10:**
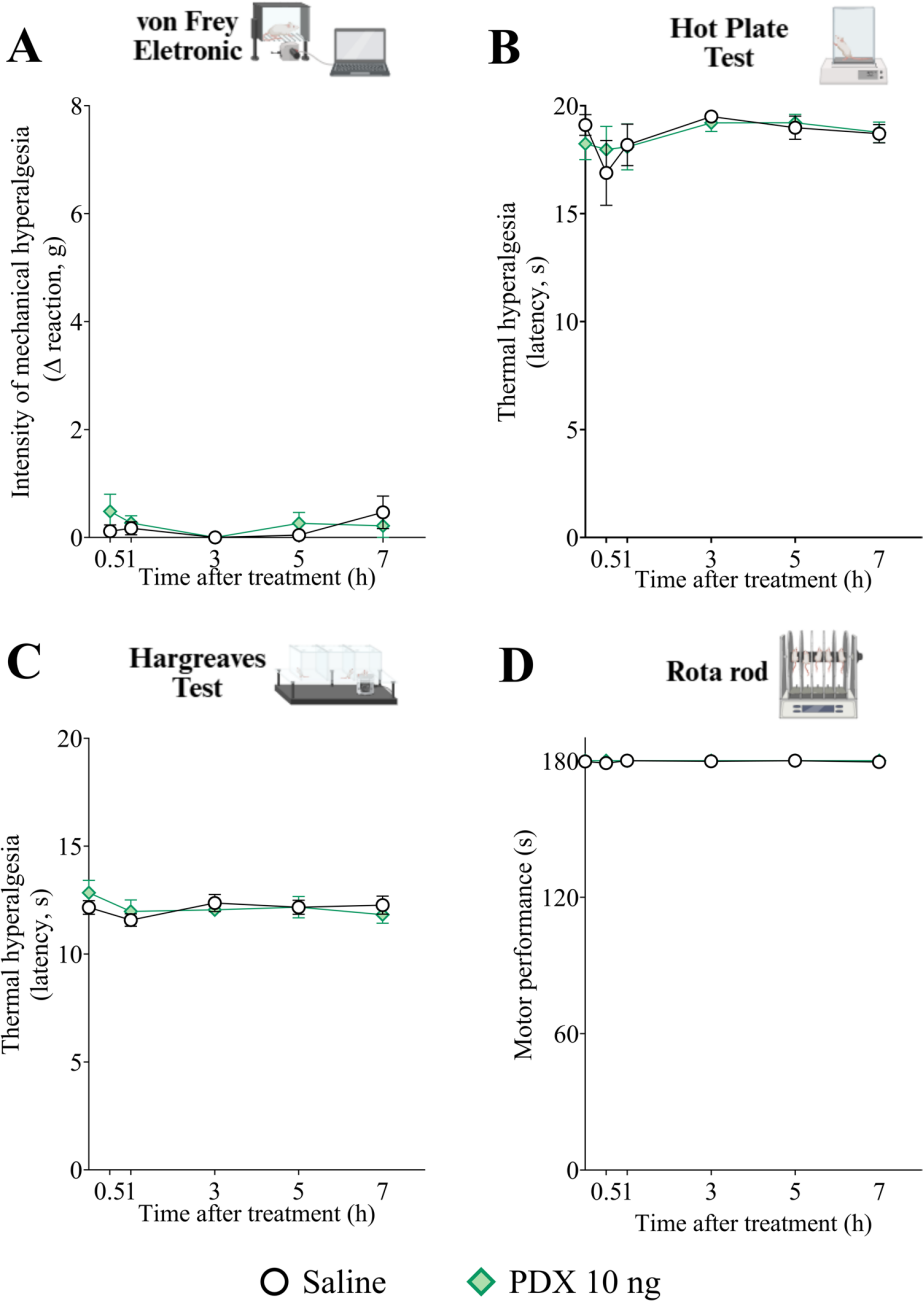
PDX does not alter baseline mechanical sensitivity, heat sensitivity and motor function in naïve mice. The mechanical sensitivity (**A**), determined by von Frey electronic method, heat sensitivity (**B**, **C**), determined by hot plate method (**B**) and Hargreaves Test (**C**), and motor function (**D**), determined by rotarod test, were evaluated 1 h after the treatment with PDX (10 ng, 100 µL, i.p.) or vehicle (sterile saline, NaCl 0.9%, 100 µL, i.p.) over 7 h. Results are expressed as mean ± SEM; n = 6 mice per group per experiment (*P < 0.05 vs saline group [two-way ANOVA followed by Sidak’s post hoc test])

Aimed to verify that the reduction in nociceptive behavior provided by PDX treatment is derived from an antinociceptive effect, and not from an anesthetic or muscle relaxation effect that could avoid the animals to respond to a stimulation, we evaluated whether PDX (10 ng), in the absence of KO_2_ stimulus, would cause changes in mechanical sensitivity, thermal sensitivity, and motor function (Fig. 10).

Mice were treated with PDX (10 ng, 100 µL, i.p.) or vehicle (0.7% ethanol in sterile saline, NaCl 0.9%, 100 µL, i.p.) 1 h before the start of the experiments. The mechanical sensitivity was assessed using the electronic von Frey method. PDX did not generate change in mechanical sensitivity over all the analysis time-points (Fig. 10A). The thermal sensitivity was determined using the hot plate method and the Hargreaves test. PDX also did not produce any change in the heat sensitivity in either of the analysis periods (Fig. 10B, C). Finally, motor function was analyzed using the rotarod apparatus. PDX did not cause any motor function deficits in the mice at either of the analysis times (Fig. 10D).

Thus, these results confirmed that the administration of PDX provides an antinociceptive effect, rather than an anesthetic or muscle relaxation action, since the baseline threshold of mechanical (Fig. 10A) and thermal sensitivity (Fig. 10B, C), as well as the motor activity capability (Fig. 10D), were not altered upon treatment with the drug.

## Discussion

We report that PDX reduced KO_2_-induced pain and inflammation. PDX i.p. treatment attenuated mechanical and thermal hyperalgesia, mechanical allodynia, unbalanced weight distribution in the hind paws, overt pain-like behaviors, leukocyte recruitment, and histopathological changes. The reduction of these disease parameters by PDX were also accompanied by diminished production of pro-inflammatory/ pro-nociceptive cytokines (TNF-α and IL-1β) and oxidative stress. Corroborating these data, we demonstrated that PDX treatment inhibited the activity of TRPV1^+^ nociceptive DRG neurons. Moreover, we demonstrated that PDX confers an antinociceptive effect, and not an anesthetic or muscle relaxation action, which was observed through the mechanical and thermal sensitivity analysis and the motor performance evaluation in non-inflamed mice.

Pain is one of the cardinal signs of inflammation and, when left untreated, can lead to reduced quality of life for patients [54]. Current therapies include the use of anti-inflammatory and antinociceptive drugs, such as NSAIDs and opioids. However, several studies have highlighted the problem of long-term use of these pharmacological agents, which includes multiple side effects, such as addiction, high cost, and toxicity, resulting in limited efficacy [29]. Therefore, novel effective therapeutic strategies for analgesic management are essential for acute inflammatory pain.

Treatment protocols employing NSAIDs can result in gastric, hepatic, and renal damage, which is associated with renal damage and constitutes a significant limitation to their clinical application [55]. In contrast, experimental evidence indicates that different members of the SPM class present a safe pharmacological profile, without inducing these types of lesions, in both acute and chronic models. Saraiva-Santos et al. [56] demonstrated that the administration of lipoxin A4 (LXA_4_; 10 ng, i.p.) every two days for 30 days in a TiO_2_-induced arthritis model did not alter gastric myeloperoxidase (MPO) activity or serum levels of ALT, AST, urea, and creatinine, indicating an absence of gastric, hepatic, or renal damage. Similarly, Li et al. [57] observed that systemic administration of RvD1 (100 or 300 ng, i.p.) for 14 days in a nonalcoholic steatohepatitis (NASH) model reduced plasma levels of ALT and AST. Zaninelli et al. [58] demonstrated that a single dose of RvD1 (3 ng, i.p.) did not induce hepatic or renal alterations in an acute model evaluated 15 h after intra-articular stimulation with MSU. Furthermore, sepsis models using PDX [59], resolvin D5 (RvD5) [60], and maresin 1 (MaR1) [61] demonstrated a reduction in these biomarkers, corroborating the safety profile of this class of mediators. In our experimental context, acute treatment with PDX (10 ng, i.p.) did not promote an increase in plasma levels of ALT, AST, creatinine, and urea, or in gastric MPO activity, reinforcing the absence of evidence of systemic toxicity in the experimental conditions evaluated.

KO_2_ injection induces inflammation and pain by the release of superoxide anion [9, 19, 26]. Superoxide anion activates endothelial cells increasing vascular permeability thereby contributing to leukocyte recruitment [11, 62, 63]. In turn, the neutrophils and macrophages that migrate to the inflammatory focus, stimulated by the inflammatory mediators (e. g., histamine, IL-1β, and TNF-α) start to produce high levels of superoxide anion via NOX2. These immune cells secrete additional pro-inflammatory cytokines into the microenvironment, amplifying the inflammation and contributing to the hyperalgesia development [3, 64, 65]. In addition, Yamacita-Borin et al. [9] demonstrated the key role of TNF-α in the KO_2_-induced inflammatory pain mechanism. In this paper, genetic deletion or pharmacological blockage of TNFR1 reduces pain and inflammation parameters, attenuating hyperalgesia, overt pain-like behaviors, neutrophil migration, and oxidative stress.

Moreover, several studies have highlighted the importance of leukocyte recruitment, especially neutrophils, in the genesis of inflammatory pain [66–69]. Guerreiro et al. [66] demonstrated, in a model of zymosan tibio-tarsal inflammation, that the peak of hyperalgesia that occurs between 7 and 24th h after the stimulus coincides with the maximum migration of neutrophils, highlighting the importance of these immune cells for the development of pain. In agreement with those results, the use of fucoidan (L-selectin inhibitor) and an anti-neutrophil antibody in zymosan arthritis model protects neutrophil migration and consequently reduces pain [67]. Cunha et al. [68] demonstrated that blocking neutrophil migration by the use of fucoidan inhibits pain caused by carrageenan, preventing neutrophil recruitment while neutrophils are a source of PGE_2_ production in response to IL-1β at the inflammatory foci. Similarly, the use of DF 2163, a non-competitive allosteric inhibitor of CXCR1/2, reduces neutrophil influx and attenuates mechanical hyperalgesia caused by CXCL1, carrageenan, and LPS [69]. Furthermore, the presence of cytokines such as TNF-α and IL-1β at the inflammatory site potentiates neutrophil activity, contributing to pain. In addition, Geraghty et al. [70] demonstrated that depletion of macrophages, mainly of the M1 phenotype, in arthritic mice reduces mechanical allodynia and knee hyperalgesia. Macrophage depletion leads to a significant reduction in Ly6G^+^ neutrophils recruitment, which is a mechanism accounting to reducing the observed pain [70]. Indeed, macrophages produce IL-1β and contribute to neutrophil recruitment as part of their role in pain induction [70–72]. In the KO_2_ model, PDX treatment inhibited the superoxide anion-triggered recruitment of neutrophils and macrophages to the plantar tissue, as well as decreased pro-inflammatory cytokine (TNF-α and IL-1β) levels. Therefore, the anti-inflammatory effect of PDX might be a contributing mechanism to the observed antinociceptive. In fact, several studies have investigated the anti-inflammatory effects of PDX in inflammatory models. Stein et al. [72] showed that PDX treatment in a postoperative ileum model reduces neutrophil infiltration in mice. In contrast, genetic deletion of 12/15 LOX leads to reduced PDX levels and, consequently, an increase in neutrophil infiltration in the postoperative period, highlighting its endogenous relevance in controlling the recruitment of polymorphonuclear leukocytes [72]. Similarly, in LPS or hyperoxia-induced lung injury models [32, 33, 73], PDX administration reduces endothelial activation and pulmonary edema, resulting from lower vascular permeability and inhibition of neutrophils and macrophage recruitment. The mechanisms involved in these anti-inflammatory effects include an increase of anti-inflammatory cytokines, such as IL-10, and glycocalyx components, such as heparan sulphate, and a decrease in pro-inflammatory cytokine (TNF-α, IL-1β, and IL-6) and chemokine (MCP-1, MIP-2, and MIP-1α) levels [32]. Furthermore, a recent study published during the peer review process of the present manuscript showed that PDX reduces postoperative pain induced by tibial fractures by actively modulating the inflammatory response. Li et al. [31] demonstrated that PDX requires binds to the GPR37 receptor on macrophages to promote the activation of the Ca^2+−^dependent GPR37–Gi/o–Gβγ–PI3K/Akt axis, resulting in increased phagocytosis, enhanced efferocytosis of apoptotic neutrophils in vitro and at the fracture site in vivo, as well as increased IL-10 levels and reduced IL-1β and TNF-α levels, leading to the conclusion that PDX switched macrophage profile to a pro-resolving phenotype [31]. Therefore, our data corroborate these previous works, highlighting the efficient anti-inflammatory activity of PDX.

Inflammation and pain, including those triggered by superoxide anion, can be mitigated by antioxidant molecules, such as quercetin [11, 74], curcumin [21], tempol [9, 75], or apocynin [9], emphasizing the relevance of ROS in the mechanisms of development and maintenance of inflammatory pain. We demonstrated that PDX reduces superoxide anion levels, restores total antioxidant defense (as evidenced by the normalization of iron reduction levels and ABTS radical elimination in plantar tissue), and increases endogenous antioxidant systems (catalase activity and GSH content). Correspondingly, other studies using PDX, especially in vitro, have demonstrated similar antioxidant mechanisms. Liu et al. [38] evaluated in vitro the PDX antioxidant effect in PMA-and fMLF-induced neutrophils. This study demonstrated that PDX inhibits NADPH oxidase activation, resulting in lower superoxide anion generation, as well as reducing MPO release and inhibiting COX-1 and COX-2 activity, limiting the oxidative and pro-inflammatory capacity of these immune cells. In an H_2_O_2_-induced endothelial cell culture model, PDX promotes an increase of SOD2 (MnSOD) and catalase expression, attenuating oxidative stress [36]. In addition, Piao et al. [37] also demonstrated that PDX decreases RNS generation. The pre-incubation of rat chondrocytes in vitro with PDX in lessened iNOS expression and NO levels and diminished PGE_2_ production. Finally, in a model of type 1 (DM1) and type 2 (DM2) diabetes mellitus, PDX increases catalase, SOD, and GSH levels, leading to a decrease in LPO and NO plasmatic levels [39].

Few works investigated the PDX antinociceptive effect. Fonseca et al. (2017) [76] evidenced that i.pl. pretreatment with PDX (70, 285 and 570 pmol) in a carrageenan-induced inflammatory pain model did not reduce mechanical hyperalgesia. This prior evidence indicates that the present results of PDX inhibiting inflammatory pain are contra-intuitive or at least not entirely expected. In turn, in a model of lumbar radicular pain from disc herniation [77], PDX i.t. post-treatment reduces mechanical and thermal hyperalgesia parameters. The antinociceptive effect observed in this work seems to be due to the decrease in IL-6 and IL-1β, and the increase in TGF-β promoted by PDX. Recently, Li et al. [31] demonstrated that both intravenous (i.v.) pre-treatment and post-treatment with PDX (100 ng) significantly reduced mechanical allodynia, thermal hyperalgesia, cold allodynia, and spontaneous pain in male and female mice, showing potency superior to neuroprotectin 1 (NPD1), maresin 1 (MaR1), resolvin D5 (RvD5), docosahexaenoic acid (DHA), gabapentin, dexamethasone, and meloxicam. PDX activates macrophage GPR37, signaling via Gi/o protein with subsequent activation of the Ca^2^⁺-dependent PI3K/Akt pathway, which increases phagocytosis and efferocytosis of apoptotic neutrophils, inhibits pro-inflammatory cytokines levels, and induces IL-10 generation, promoting the reprogramming of the inflammatory microenvironment. Moreover, ex vivo calcium imaging in naïve DRG neurons demonstrated that PDX reduces spontaneous neuronal hyperactivity and attenuates TRPA1- and TRPV1-mediated responses. The observed antinociceptive effect of PDX occurs via neuronal GPR37-mediated signaling that inhibits TRP neuronal signaling. PDX also reduced the TRPA1 neurogenic inflammation unveiling the interference in neuroimmune communication. Thus, systemic treatment with PDX likely involves neuroimmune and neuron-intrinsic pathways to attenuate peripheral sensitization and DRG hyperexcitability. Consistently, our results corroborate recent studies [31, 77], as PDX (10 ng) reduced mechanical hyperalgesia, thermal hyperalgesia, mechanical allodynia, overt pain-like behaviors, and imbalance in weight distribution by neuronal mechanism.

The absence of antinociceptive activity of PDX in the work of Fonseca et al. [76] contrasts with the results obtained by Zhao et al. [77], Li et al. [31] and our research group, requiring a careful analysis of the methodological and biological factors involved in this divergence. When comparing the experimental protocols used by Fonseca et al. [76], Zhao et al. [77], Li et al. [31], and our group, we can assume that the apparent discrepancies regarding the antinociceptive efficacy of PDX are not contradictory, but reflect differences in the pathophysiological context, routes and administration schedules, timing of intervention, and the types of methods used to assess nociceptive behaviors.

Fonseca et al. [76] used a model of acute inflammatory pain induced by carrageenan in Swiss mice. This model is characterized by acute exudative inflammation, driven by the rapid production of primary inflammatory mediators such as prostaglandins, histamine, serotonin, and bradykinin, responsible for nociceptive sensitization. The study demonstrates that a 10-min i.pl. pretreatment with PDX does not reduce mechanical hyperalgesia as assessed over 6 h after i.pl. carrageenan stimulation (300 µg/paw). In this experimental context, the antinociceptive ineffectiveness may be explained by some factors, such as the experimental model, the short pretreatment interval, and the chosen route of administration. Local injection, restricted to the immediate tissue microenvironment and a short time window, may not allow for more robust immune modulation compared to systemic distribution protocols. Another point is that the study was limited to only one pain parameter, mechanical hyperalgesia, not exploring the ability of PDX to reduce thermal hyperalgesia, mechanical or cold allodynia, and spontaneous pain, for example. Thus, there are many subtle protocol differences, but no clear explanation for the lack of PDX activity in the carrageenan model.

In contrast, in the work of Zhao et al. [77], i.t. post-treatment reduces mechanical and thermal hyperalgesia in a model of chronic neuropathic pain induced by lumbar radicular compression in Sprague–Dawley rats. This experimental model is characterized by neuroinflammation, glial activation, and central sensitization, which are maintained for one week. In this context, factors such as the route of administration and the treatment regimen may have favored the antinociceptive action. I.t. administration ensures direct access to the dorsal horn of the spinal cord, favoring the modulation of central nociceptive circuits, which is essential in this pain model. Furthermore, the treatment is administered once daily for three days, which may have contributed to a prolonged effect of PDX, allowing the activation of pro-resolving signaling pathways that require more time to promote transcriptional and phenotypic changes. In turn, Li et al. [31] demonstrated that a single i.v. administration of PDX reduces mechanical and thermal hyperalgesia, cold allodynia, and spontaneous pain in a postoperative pain model induced by tibial fracture in C57BL/6 mice. This model is characterized by significant tissue damage, persistent neuroinflammation, and active neuroimmune interaction. In this context, the chosen experimental model and route of administration may have contributed to the observed antinociceptive effect, and also that the pathophysiological mechanisms of the disease were amenable to PDX changes in cellular phenotypic changes.

Finally, our KO_2_ model (30 µg i.pl., 7 h) also differs substantially from the carrageenan model. Although both are acute, KO_2_ induces pain predominantly via oxidative stress, with ROS generation and activation of redox-sensitive pathways. The carrageenan inflammation depends on TLR4 and inflammasome activation [78]. PDX (10 ng, i.p., 1 h before) reduced mechanical and thermal hyperalgesia (hot plate and Hargreaves test), mechanical allodynia, spontaneous pain (flinches, licking, and abdominal writhings), and changes in weight distribution (SWB). Unlike Fonseca, we used a systemic administration route and a longer pre-treatment interval, which may have resulted in greater bioavailability and time to allow the modulation of peripheral immune compartments. Furthermore, we evaluated different parameters of nociception, which increased the sensitivity to detect the effects of PDX. Taken together, these studies [31, 76, 77] indicate that PDX does not function as a conventional anti-inflammatory analgesic that, for instance, directly blocks prostaglandin synthesis. On the contrary, these data collectively support the idea that PDX interferes with the activation, function and phenotypes of immune cells and [31, 77 and present data], nociceptive neuron plastic changes and activation [31 and present data], and also neuroimmune interactions [31] depending on the disease contexts.

Moreover, we evidenced that PDX (10 ng, i.p.) treatment, in the absence of KO_2_ injection, does not alter the basal mechanical and thermal sensitivity and motor performance. These data show that the inhibitory effect on the nociceptive behaviors provided by PDX is an antinociceptive action, excluding possible anesthetic or muscle relaxation properties that could prevent the animal response to the mechanical and thermal stimulation or ability to walk in the rota-rod test [79]. Jointly, these data demonstrate that PDX reduces nociceptive behaviors through immune cell and neuronal mechanisms.

ROS and RNS can activate nociceptors directly and indirectly, contributing to peripheral and central activation and sensitization. The ROS generated during the inflammation react directly on intracellular residues of ion channels for neuronal activation, such as TRPV1, generating alterations that facilitate the opening of these channels [22]. In particular, nociceptive neurons in the periphery significantly express TRPV1, a channel that is sensitive to the redox state and essential for the genesis of pain and inflammation. ROS interact with cysteine residues distributed along the TRPV1 structure or, in the case of RNS, promote the nitration or nitrosylation of these residues [13, 22, 23]. The redox alterations facilitate nociceptive neuron activation via the influx of Ca^2+^ through TRPV1, contributing to inflammatory pain. Indirectly, ROS also stimulates the pro-inflammatory mediators (e.g.,TNF-α, IL-1β, IL-6, IL-8 and PGE_2_) production [80]. In turn, these molecules bind to their cognate receptors expressed by the nociceptive neurons, activating signaling pathways such as NF-κB, MAPK, and PLC, which result in the activation of PKA and PKC, capable of phosphorylating channels such as TRPV1, facilitating neuronal activation [8, 14, 22]. Thus, considering the TRPV1 relevance in inflammatory pain, molecules that control the redox state of the inflammatory microenvironment reduce the activation of nociceptive ion channels and, consequently, diminish neuronal activation, resulting in analgesia.

To investigate TRPV1 as a potential PDX target, we performed a calcium imaging assay. As a control for the neuronal population, we used stimulation with capsaicin, a TRPV1 agonist, which was used to demonstrate neuronal responsiveness to the stimulus and to identify TRPV1^+^ DRG neurons. Therefore, we demonstrated that in cultured primary sensory DRG neurons of mice that received KO_2_ in vivo, there was a higher basal level of calcium in TRPV1^+^ neurons (capsaicin-responsive) than in the DRG of non-stimulate mice. In addition, KO_2_ also enhanced TRPV1 staining and TRPV1 co-stained with nuclear p-NF-κB p65 in DRG neurons. The p-NF-κB p65 staining was applied as a surrogate marker of cellular activation indicating that TRPV1^+^ neurons were less activated upon PDX treatment [58]. Thus, the combined data of calcium influx and immunofluorescence allow concluding that PDX reduced KO_2_ activation of TRPV1^+^ nociceptive DRG neurons. Other SPMs, such as MaR1 [81], MaR2 [82], LXA_4_ [56] and PD1 [83], reduce inflammatory pain by inhibiting the expression and/or activity of TRPV1 in DRG neurons. Recently, Li et al. [31] demonstrated that PDX promotes an antinociceptive effect in a postoperative fracture pain model in mice via GPR37 activation. In addition, this study demonstrated that i.v. treatment with PDX (100 ng) induces polarization of macrophages toward the pro-resolving phenotype and modulates the activity of TRPA1 and TRPV1 channels, reducing the activation of DRG neurons, which explains the antinociceptive action observed. These findings, in a chronic pain model, corroborate our results, since we demonstrated the ability of PDX to modulate TRPV1 activation in DRG neurons, but in a context of acute pain triggered by ROS. Thus, our work is contributing to consolidate a concept on how SPM exert their antinociceptive mechanisms. These data also demonstrate that the antinociceptive effect of PDX involves a reduction in pro-inflammatory cytokines, ROS, and TRPV1 activity. The reduction of pro-inflammatory cytokines (TNF-α and IL-1β) and ROS by PDX can account to reduce TRPV1 activation and expression, which are up-regulated by cytokines and ROS.

Our study demonstrated the anti-inflammatory and antinociceptive activity of PDX in a superoxide anion-induced inflammatory pain model, and it is relevant to consider some limitations of our experimental design and the analyzes performed. One of these limitations refers to the time frame of the antinociceptive effect of PDX analyzes, evaluated up to the 7th hour after induction of the inflammatory pain model. Our results showed that pretreatment with PDX consistently reduced pain parameters up to the 7th hour after stimulation. However, no analyzes were performed after the 7th hour to verify the duration of the antinociceptive effect of PDX, determining how long the nociception would be attenuated. Thus, although our design was structured to investigate the acute phase of the nociceptive response, as previously standardized [19], this temporality prevents the continued evaluation of the duration of PDX activity. Our experimental design does not allow us to properly correlate the reduction in leukocyte recruitment with the observed antinociceptive effect. This is because specific protocols were not performed to mechanistically test whether antinociception would be diminished by presence of activated neutrophils and macrophages in the plantar tissue. Finally, the toxicity assays were conducted in an acute setting, with a single administration of PDX and evaluation at an early time point (8 h after treatment), without repeated administration protocols for prolonged periods, or detailed histological analysis of all possible target organs. Although no biochemical changes compatible with hepatic, renal, or gastric damage were observed, and comparisons were made with samples from protocols in which NSAIDs induce such lesions as a form of positive controls, a proper comparison should use similar protocols of treatment for the compared compounds. Therefore, it is not possible to claim superiority or equivalence in safety compared to this class of anti-inflammatory drugs, but only to indicate that, in the acute model used, PDX did not induce these types of damage.

**Fig. 11:**
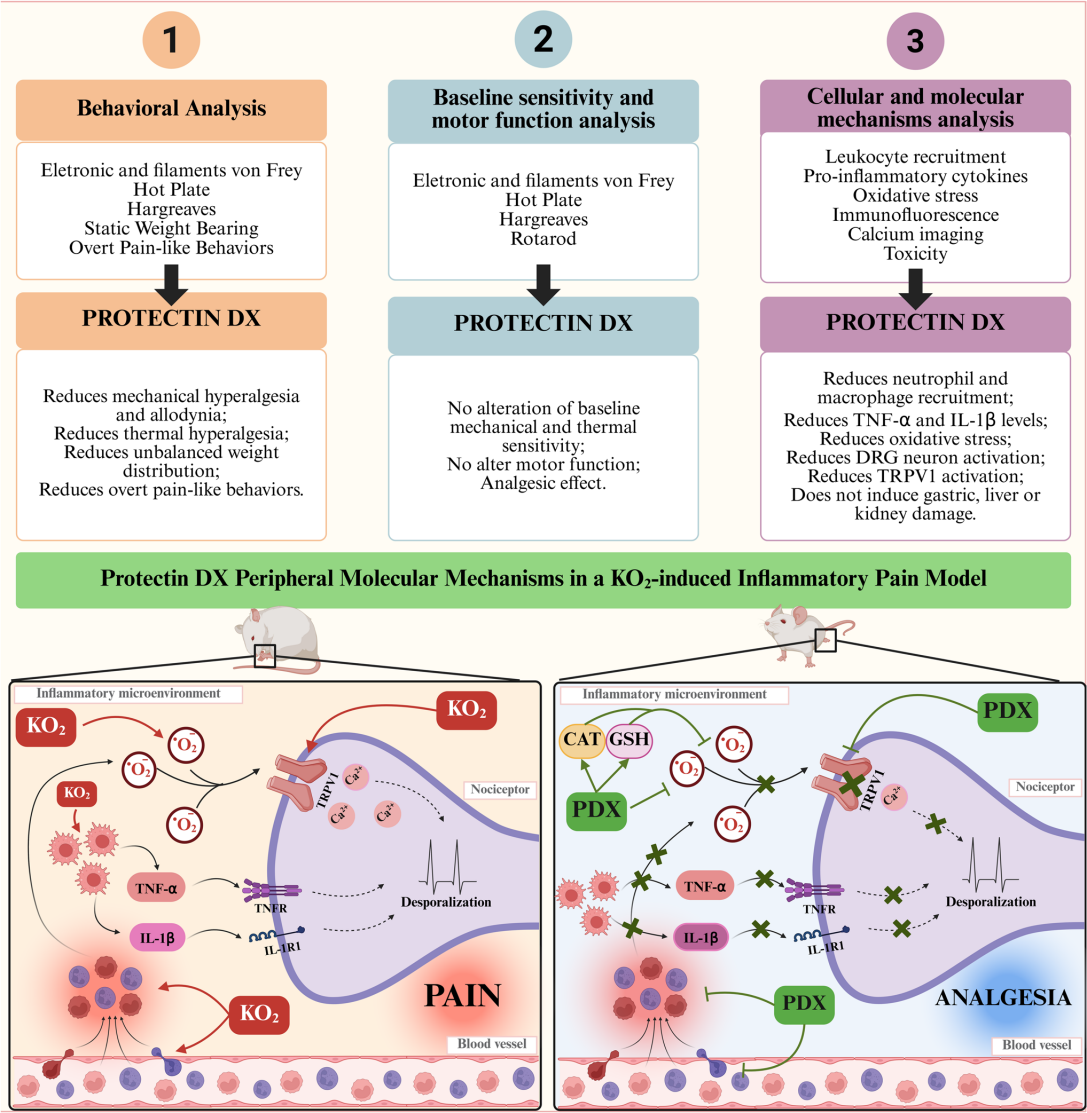
induction of

Therefore, our research demonstrated for the first time, in an acute superoxide anion-triggered inflammatory pain model in vivo, that PDX inhibits pro-inflammatory cytokines and ROS production, increases levels of antioxidant agents, reduces leukocyte recruitment, and TRPV1^+^ nociceptive neuron activation, providing a reduction in evoked and non-evoked nociceptive behavior parameters triggered by the superoxide anion donor injection (illustrated in Fig. 11). Thus, we expanded the understanding of PDX anti-inflammatory, induction of antioxidant response, and antinociceptive mechanisms unveiling a hitherto unrecognized neuronal antinociceptive mechanisms of PDX in the context of nocifensive ROS responses.

## Supplementary Information

Below is the link to the electronic supplementary material.

Supplementary file1 (PDF 298 kb)

Supplementary file2 (PDF 688 kb)

## Data Availability

Data will be made available on request.
